# Desmosomes as Signaling Hubs in the Regulation of Cell Behavior

**DOI:** 10.3389/fcell.2021.745670

**Published:** 2021-09-23

**Authors:** Lisa Müller, Mechthild Hatzfeld, René Keil

**Affiliations:** Department for Pathobiochemistry, Institute of Molecular Medicine, Martin Luther University Halle-Wittenberg, Halle, Germany

**Keywords:** desmosomes, proliferation, differentiation, barrier function, inflammation, EGFR, IGF1R, Hippo signaling

## Abstract

Desmosomes are intercellular junctions, which preserve tissue integrity during homeostatic and stress conditions. These functions rely on their unique structural properties, which enable them to respond to context-dependent signals and transmit them to change cell behavior. Desmosome composition and size vary depending on tissue specific expression and differentiation state. Their constituent proteins are highly regulated by posttranslational modifications that control their function in the desmosome itself and in addition regulate a multitude of desmosome-independent functions. This review will summarize our current knowledge how signaling pathways that control epithelial shape, polarity and function regulate desmosomes and how desmosomal proteins transduce these signals to modulate cell behavior.

## Desmosome Composition

Desmosomes are cell-cell contacts that mediate strong cell-cell adhesions to guarantee tissue integrity under mechanical stress. Accordingly, they are enriched in tissues that experience recurrent mechanical stress, such as the keratinocytes of the skin, and cardiomyocytes in the heart. Desmosomes contain two types of cadherins, desmogleins (DSG1-4), and desmocollins (DSC1-3) that are expressed in a tissue- and differentiation-specific pattern. DSG/DSC heterodimers represent the fundamental adhesive unit of desmosomes (Harrison et al., 2016). Their cytoplasmic domains bind to plakoglobin (JUP alias PG) and plakophilins (PKP1-3). Like the desmosomal cadherins, PKPs reveal tissue- and differentiation-dependent expression patterns. These proteins interact with desmoplakin (DSP) to link the desmosomes with the keratin filament network, which is essential to provide tensile strength. In contrast to adherens junctions (AJ), desmosomes can undergo a process of “maturation,” rendering them calcium-independent also referred to as hyperadhesive (Garrod and Tabernero, 2014; Broussard et al., 2015; Najor, 2018).

Changes in desmosome composition during keratinocyte differentiation determine distinct characteristics of the desmosomes: basal keratinocytes express the desmosomal cadherins DSC2/3 and DSG2/3, whereas the expression of DSC1 and DSG1/4 is restricted to differentiated cells. Desmosomes in the basal layer need to be dynamic to allow for proliferation which is a prerequisite for tissue regeneration and remodeling. In contrast, the differentiated cells of the suprabasal layers provide stable cell-cell adhesion to secure cornified envelope formation and protect the epidermis from chemical and mechanical stresses (Green et al., 2019). These distinct requirements correlate with distinct characteristics of the desmosomal cadherins: in a systematic approach to determine the interactions among the desmosomal cadherins by surface plasmon resonance, the strongest binding was observed between the suprabasal cadherins DSG1/DSC1 and DSG4/DSC1, whereas the basally expressed DSG3/DSC3 revealed the weakest binding (Harrison et al., 2016). Similarly, PKP expression patterns in the skin correlate with more dynamic (PKP2, PKP3) or stable and calcium-independent desmosomes (PKP1) (Keil et al., 2016; Fülle et al., 2021). Moreover, PKP isotype expression controls desmosome size: whereas loss of PKP1 correlated with sparse and small desmosomes and impaired adhesion in human and mouse skin, elevated PKP1 levels yielded larger desmosomes (McGrath et al., 1997; Kowalczyk et al., 1999; Hatzfeld et al., 2000; South et al., 2003; Rietscher et al., 2016). In contrast, loss of PKP3 did not provoke an obvious adhesion defect (Sklyarova et al., 2008). Tricellular junctions are different from bicellular (or lateral) junctions adding another level of complexity. These regions are hotspots of tension and recent studies have uncovered a role of tricellular junctions in the regulation of the epithelial cell division orientation, which is essential for morphogenesis and the maintenance of tissue polarity (Bosveld et al., 2016; Nestor-Bergmann et al., 2019; Higashi and Chiba, 2020). In keratinocytes, PKP3 accumulated at tricellular contacts, whereas PKP1 was excluded from these regions (Keil et al., 2016; Rietscher et al., 2018). So far, the composition of PKP3-containing tricellular junctions remains elusive. Collectively, these data indicate that isoform expression has a considerable influence on desmosome dynamics, stability and resistance to force and appears well-suited to adapt desmosomes to their changing environment that requires plasticity as well as stability.

Beyond structural functions preserving mechanical resistance of tissues, desmosomal components are also indispensable for intracellular signaling. As extensively described in various recent reviews (Najor, 2018; Costa et al., 2020; Egami et al., 2020; Chen et al., 2021; Lee and McGrath, 2021; Mohammed and Chidgey, 2021) numerous diseases of the skin and/or the heart arise if desmosomal proteins are compromised. These disorders show a plethora of clinical manifestations and are often accompanied by dysregulated proliferation and/or inflammation. Moreover, several knockout and transgenic animal models for desmosomal proteins (Supplementary Table 1), support the role of desmosomes as signaling hubs that regulate cellular behavior in various tissues. To illustrate the close connection between structural and signaling functions of desmosomes, we focus here on desmosome in epidermal keratinocytes and their regulation by signaling pathways that affect proliferation, survival, differentiation, and inflammation as well as the impact of desmosomal proteins on these pathways. For detailed information on assembly of desmosomes and their interplay with tight junctions, adherens junctions and gap junctions as well as on their role in the heart we refer to recent reviews (Patel and Green, 2014; Hatzfeld et al., 2017; Piven and Winata, 2017; Garcia et al., 2018; Green et al., 2019; Costa et al., 2020; Gerull and Brodehl, 2020).

## Regulation of Desmosomal Functions

**FIGURE 1:**
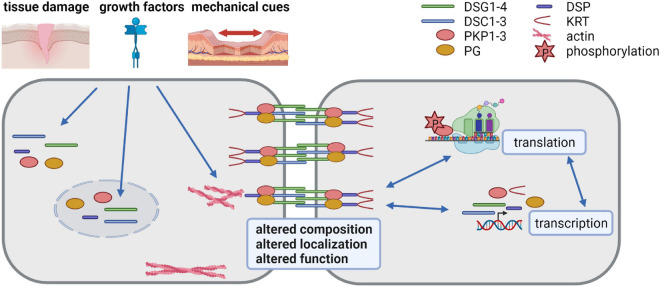
Desmosomes as dynamic structures *(created with biorender.com).* Desmosomes are composed of the desmosomal cadherins desmoglein (DSG) 1-4 and desmocollin (DSC) 1-3, the armadillo family proteins plakoglobin (PG) and plakophilin (PKP) 1-3 and the plakin family protein desmoplakin (DSP) that anchors keratin filaments. Their expression is tightly regulated at transcriptional, posttranscriptional, translational and posttranslational level. Tissue damage, growth factors and mechanical cues affect desmosomes by altering their composition, localization and function. Thus, the dynamic modulation of desmosomes is crucial for cells adapting to a changing environment.

Desmosome composition, size and number vary among tissues and among the individual layers of the epidermis and can adapt to environmental insults. The molecular mechanisms responsible for the differential expression of desmosomal proteins and the regulation of their diverse functions are only incompletely understood. Here we discuss the progress that has been made to decipher the regulation of desmosome composition and function at the transcriptional, posttranscriptional, and posttranslational levels (summarized in Figure 1).

### Transcriptional Regulation

So far, the interplay between transcription factor networks and context-dependent stimuli that control desmosome gene transcription and isotype expression remain incompletely understood. In the epidermis, differential expression and/or activity of transcription factors would be expected to regulate the differentiation-dependent expression of genes including desmosomal genes. Several transcription factors are known to control stratification and barrier formation. The transcription factor tumor protein 63 (Tp63) is necessary for both, epidermal stem cell self-renewal and differentiation, whereas CCAAT/enhancer-binding protein (C/EBP) α/β, Kruppel-like factor (KLF) 4, and grainyhead-like (GRHL) 3 promote differentiation (Segre et al., 1999; Ting et al., 2005; Truong et al., 2006; Senoo et al., 2007; Lopez et al., 2009; Sen et al., 2012). Tp63 regulates a subset of desmosomal genes including DSG1, DSC3, and DSP which were significantly reduced by mutant Tp63. Chromatin immunoprecipitation (ChIP) and transactivation assays indicated that Tp63 directly controls the transcription of these genes (Ferone et al., 2013).

Aiming to understand the processes underlying the differential expression of *DSC* genes in the epidermis, Smith et al. (2004) isolated the *DSC1* and *DSC3* 5′-flanking DNA regions and analyzed their activity in primary keratinocytes. They found differential regulation of *DSC* genes by C/EBP family members: C/EBPα activated *DSC1* expression while C/EBPβ promoted *DSC3* expression. In contrast, C/EBPδ supported the expression of both *DSC* genes. Analysis of the upstream sequences of *DSG* genes revealed GC-rich regions and consensus binding sites for transcription factors activator protein 1 and 2 (AP-1, AP-2) (Adams et al., 1998). Given that AP-1 is regulated by growth factor signaling *via* mitogen-activated protein (MAP) kinases, by serum response factor (SRF) and by mechanical stimuli (Kim et al., 2018; Yeung et al., 2018), it is well-suited to adapt desmosome composition and adhesive function to environmental cues.

KLF4 is essential for barrier acquisition in agreement with its high expression in the differentiating layers of the epidermis (Segre et al., 1999). KLF4 upregulated the expression of the desmosomal proteins DSP, DSG1a, and DSG1b (Swamynathan et al., 2011), whereas KLF5 expression was shown to correlate with DSG2 transcript levels in colon cells (Liu et al., 2017). Another factor that participated in the maintenance of the skin barrier is the transcription factor GRHL1. GRHL1 regulated the expression of DSG1 in suprabasal layers of the epidermis (Mlacki et al., 2014). GRHL1−binding sites were detected in the proximal *DSG1* promoters, whereas no such consensus sites were found in the basally expressed *DSG2* and *DSG3* genes, or in any of the *DSC* genes. These data suggest that KLF4 and GRHL1 are involved in the differentiation-dependent activation of suprabasal DSG1 transcription. GRHL3-deficient mice exhibited a defective skin barrier and wound repair. Desmosomal genes were not described as direct targets although their expression may be modulated by the GRHL3 targeted transcription factors KLF4, OVO-like transcriptional repressor 1 and OVO-like zinc finger 2 (OVOL1, OVOL2) (Ting et al., 2005; Boglev et al., 2011; Gordon et al., 2014).

Several additional transcription factors have been shown to modulate desmosomal gene expression. These include among others the epithelial to mesenchymal transition associated transcription factors zinc finger E-box binding homeobox 1 and 2 (ZEB1, ZEB2) as well as snail family transcriptional repressor 1 and 2 (SNAI1, SNAI2) which repressed DSG2, DSG3, DSC2, PG, DSP and PKP1, PKP2, and PKP3 expression. Moreover, mechanical forces act through the actin cytoskeleton to regulate the Hippo and SRF pathways, which both affect desmosomal gene expression. ChIP sequencing identified DSG1, DSC1-3, DSP, PKP1, PKP2, and PG as putative targets of the Hippo effectors TEA domain (TEAD) transcription factors (Liu et al., 2016). Actin regulates SRF activity by inhibiting its transcriptional coactivator myocardin-related transcription factor (MRTF). An actin/MRTF/SRF regulatory axis promoted *PKP2* gene expression (Leitner et al., 2011).

In summary, several transcription factors with specificity for distinct desmosomal genes have been identified in recent years. However, further studies are required to understand how the activity of these transcriptional regulators is coordinated to ensure spatiotemporal expression of desmosomal genes during epidermal differentiation.

### Posttranscriptional Regulation

While much of the differential gene expression is achieved at the level of transcription, the contribution of posttranscriptional events to cell-specific expression patterns has recently come into focus. For example, the coordinate synthesis of functionally related proteins can be achieved at the posttranscriptional level by the action of common regulatory molecules, such as RNA binding proteins (RBPs) and non-coding RNAs (ncRNA) (Zanzoni et al., 2019). 3′-untranslated regions (3′-UTR) are known to regulate diverse fates of mRNAs, including degradation, translation, and localization. However, although most of the desmosomal genes contain long 3′-UTRs, little is known about their posttranscriptional regulation.

#### MicroRNAs

The expression of microRNAs (miRNA) is spatiotemporally regulated in the epidermis (Yi et al., 2006) and miRNAs have been shown to control skin development by targeting mRNAs encoding critical transcription factors and components of signaling pathways. The general role of miRNAs in skin development has been studied by preventing miRNA biogenesis through depletion of Dicer or DiGeorge syndrome critical region 8 (DGCR8) (Yi et al., 2006, 2009; Ghatak et al., 2015). The epidermis specific ablation of Dicer resulted in altered keratinocyte differentiation with increased apoptosis, barrier defects, and neonatal lethality in the knockout tissue (Andl et al., 2006). An individual miRNA may interact with an entire set of genes, while the expression of a single gene may be controlled by multiple miRNAs. Accordingly, knockout or overexpression of single miRNAs can have a broad impact. Reports on the function of individual miRNAs in the skin include miR-203, which promoted differentiation and suppressed stemness of keratinocytes through the repression of Tp63 (Lena et al., 2008; Yi et al., 2008). Since Tp63 regulates the expression of several desmosomal proteins, these findings imply an indirect control of desmosomal gene expression by miR-203. miR-125b was associated with stemness through the regulation of the transcription factor GRHL1 and of DSG1a, raising the possibility that the differentiation specific expression of DSG1 is directly and indirectly controlled by miR-125 (Zhang et al., 2011). miR-29a/b directly targeted DSC2, which impaired desmosome adhesiveness in keratinocytes and induced structural alterations of epidermal desmosomes. Expression of miR-29a/b was increased upon nuclear factor erythroid 2 related factor 2 (NRF2) activation, a mediator of cellular resistance to oxidative stress (Kurinna et al., 2014). In nasopharyngeal carcinoma, upregulated miR-149 decreased PKP3 expression by direct binding to the PKP3 3′-UTR (Li et al., 2018). Taken together, so far only a few miRNAs have been identified that directly target desmosomal transcripts. However, the long 3′-UTRs of most desmosomal transcripts contain numerous putative miRNA target sites, which suggests that additional miRNAs are involved in their regulation.

#### Long Non-coding RNAs

Long non-coding RNAs (lncRNAs) are a largely uncharacterized group of ncRNAs with diverse regulatory roles in biological processes. Recent observations have elucidated roles in the control of proliferation, differentiation, and stratification of epidermal keratinocytes and in wound repair (Piipponen et al., 2020b). Anti-differentiation ncRNA (ANCR) was highly enriched in epidermal progenitor cells and downregulated during differentiation. Knockdown of ANCR led to premature epidermal differentiation with a strong upregulation of DSC1 and DSG1 which was most likely mediated by ANCR-regulated transcription factors including GRHL3, ZNF750, and KLF4 (Kretz et al., 2012). In contrast, terminal differentiation-induced ncRNA (TINCR) was upregulated during differentiation and transcription factors GRHL1 and KLF4 as well as DSC1 and DSG1 were downregulated in TINCR-depleted epidermis. At the molecular level, a TINCR-Staufen1 complex seemed to stabilize target transcripts. In agreement, Staufen1 deficient cells recapitulated the downregulation of TINCR target transcripts including DSC1 and DSG1 (Kretz, 2013). Recently, the classification of TINCR as a lncRNA has been challenged by the finding of an open reading frame and detection of the corresponding protein as a component of cornified keratinocytes (Eckhart et al., 2020).

#### RNA Binding Proteins

So far, little is known about the posttranscriptional regulation of desmosomes by RBPs. The DSP mRNA was detected in fragile X related protein 1 (FXR1) immunoprecipitates from cardiac muscle and DSP transcript and protein were upregulated in FXR1 knockout hearts. *In vitro* assays indicated that FXR1 bound directly to the DSP mRNA and repressed its translation (Whitman et al., 2011). Moreover, FXR1 formed a complex with PKP1 and PKP3 that stabilized the PKP2 mRNA in prostate cancer cells (Fischer-Keso et al., 2014). Large scale approaches based on crosslinking immunoprecipitation (CLIP) (including HITS-CLIP, PAR-CLIP, and iCLIP) have been performed to identify transcriptome-wide binding sites of RBPs. These studies identified a number of putative interactions between RBPs and mRNAs coding for desmosomal proteins (e.g., CLIPdb^1^; Yang Y. C. et al., 2015). However, these data require validation of the binding sites and examination of functional consequences.

Taken together, posttranscriptional control of desmosome composition during differentiation and stress appears to play an important role in modulating desmosome function. However, many RBPs and ncRNAs involved remain to be identified and their interplay and functional relevance need to be studied.

### Posttranslational Regulation

Posttranslational modifications (PTM) of proteins are crucial for controlling protein stability, localization, and protein interactions and play a key role in numerous biological processes. Reversible modifications include methylation, acetylation, palmitoylation, sumoylation, ubiquitylation, and phosphorylation of specific amino acid side chains. Such modifications coordinately exert dynamic control over protein function in diverse biological contexts. Desmosomal proteins and especially the desmosomal plaque proteins are highly modified by phosphorylation, which in turn is regulated by signaling cascades that are activated by growth factors, mechanical signals or cytokines (summarized in Figure 1). Here, we will focus on the roles of epidermal growth factor receptor (EGFR), insulin like growth factor 1 (IGF1) receptor (IGF1R), and Hippo signaling pathways in controlling desmosome function.

#### Epidermal Growth Factor Signaling

**FIGURE 2:**
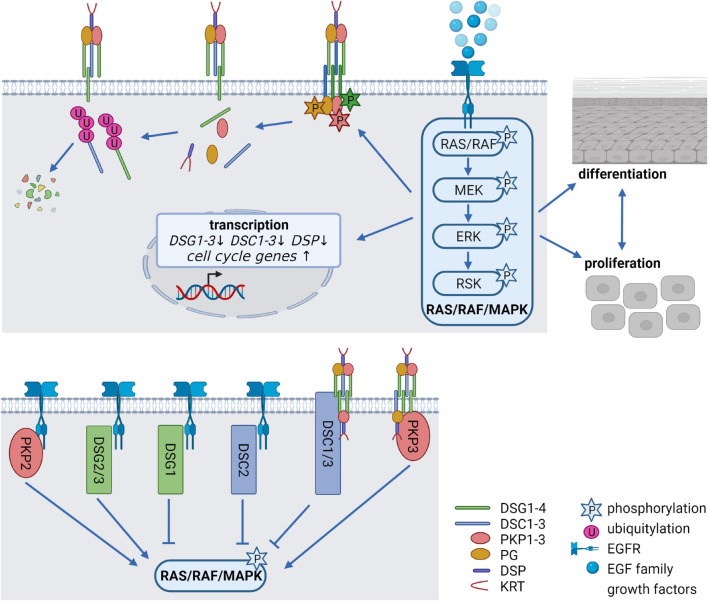
Epidermal growth factor receptor (EGFR) signaling as a critical regulator of desmosomes *(created with biorender.com)*. **(Upper panel)** Modification of desmosomes by EGFR signaling. Binding of EGF family growth factors to their receptors activates the canonical RAS/RAF/MAPK signaling pathway. Several kinases of the pathway can phosphorylate desmosomal cadherins and PKPs, thereby affecting their stability as well as their localization at the cell membrane. At the same time, EGFR signaling alleviates transcription of desmosomal genes and promotes cell cycle gene expression thereby controlling the balance between proliferation, differentiation and cell-cell adhesion. **(Lower panel)** Impact of desmosomal proteins on EGFR signaling. The desmosomal cadherins DSG1-3 and DSC2 as well as PKP2 co-localize or interact with the EGFR at the cell membrane, thereby either activating or inhibiting RAS/RAF/MAPK signaling. DSC1/3 and PKP3 affect RAS/RAF/MAPK signaling probably without interfering directly with the EGFR.

The EGFR network, comprising seven ligands and four related receptors is a critical system to regulate the balance between cell cycle progression and differentiation and its deregulation is associated with numerous human disorders, including cancer. Activation of the EGFR is induced by binding of EGF family growth factors which promotes EGFR dimerization, and subsequent activation of the canonical RAS/RAF/MAPK signaling cascade (Figure 2), the phosphatidylinositol-3-kinase (PI3K)/AKT pathway, the protein kinase C (PKC) signaling cascade, or the Janus kinase/signal transducer and activator of transcription (JAK/STAT) pathway. This allows information to be transduced from the cell surface to the nucleus, where transcription of genes responsible for proliferation, cell growth, survival, motility, adhesion, and differentiation is induced (Wee and Wang, 2017; Sabbah et al., 2020). Deletion of the EGFR as well as the deletion or overexpression of its ligands in mice indicated that adequate EGFR signaling is essential for epidermal development and homeostasis. Remarkably, anti-EGFR antibodies and inhibitors targeting the receptor, which are widely used for treating diverse cancer types, are known to cause a variety of cutaneous pathologies, including rash, dry and itchy skin, inflammation as well as nail and hair abnormalities (Lacouture, 2006; Nanba et al., 2013; Dahlhoff and Schneider, 2016). Inhibition of EGFR signaling interferes with normal epidermal proliferation, whereas overexpression and/or constitutive activation of the EGFR results in hyperproliferation and cancer (Rogers et al., 2005; Kalyankrishna and Grandis, 2006). The combined deletion of MAP kinase 1 and 2 (MAP2K1, MAP2K2 alias MEK1/2) downstream of the EGFR (see Figure 2) induced hypoproliferation, apoptosis, skin barrier defects, and ultimately death (Scholl et al., 2007). Similarly, simultaneous deletion of the MEK kinase substrates MAP kinase 3 and 1 (MAPK3, MAPK1 alias ERK1/2) revealed proliferation defects and epidermal hypoplasia, whereas the depletion of ERK1 or ERK2 alone did not disrupt epidermal homeostasis (Dumesic et al., 2009). However, the contribution of desmosomal proteins to the described effects has not been addressed. Notably, a variety of studies demonstrated numerous EGFR-dependent PTMs of desmosomal proteins, suggesting that the EGFR network is a key modulator of desmosomal functions.

Pemphigus vulgaris (PV) is an autoimmune disorder in which antibodies are directed against DSG3, resulting in severe mucosal erosions and epidermal blistering. PV antibodies induced DSG3 internalization and the internalized PV IgG/DSG3 complex colocalized with markers for endosomes and lysosomes, suggesting that DSG3 was targeted for degradation (Calkins et al., 2006). This was mediated by EGFR signaling since the EGFR was activated following PV IgG treatment and inhibition of EGFR blocked PV IgG triggered DSG3 endocytosis and loss of cell-cell adhesion. These data demonstrate a crosstalk between DSG3 and the EGFR (Bektas et al., 2013; Spindler et al., 2018) and suggest that endocytic membrane trafficking is a fundamental mechanism by which cells confer a dynamic state to cell-cell contacts. EGFR signaling also regulated desmosomes in squamous cell carcinoma (SCC) by decreasing the level and cell surface localization of desmosomal cadherins (Lorch et al., 2004). This was accompanied by phosphorylation of DSG2, and matrix-metalloprotease (MMP)-dependent shedding of the DSG2 ectodomain. Both EGFR and MMP inhibition reversed these effects. Mechanistically, the internalization of DSG2 resulted from proteolytic cleavage and release of the DSG2 extracellular domain by ADAM metallopeptidase domain 17 (ADAM17), a transmembrane protease which regulates proteolysis of many growth factor receptors and adhesion molecules (Arribas and Borroto, 2002; Klessner et al., 2009).

This raises the question, which PTMs control endocytosis. EGFR signaling induced phosphorylation of DSG2 and PG (Figure 2). The effect of DSG2 phosphorylation at serine 680 in response to EGF stimulation (Huang et al., 2016) has not been investigated in detail and the responsible kinase remains elusive. PG phosphorylation in response to EGFR activation at tyrosine residues Tyr693, Tyr724, and Tyr729 resulted in a shift from the membrane to the cytoplasm (Yin et al., 2005). Phosphorylated PG remained associated with DSG2, but did not interact with DSP (Gaudry et al., 2001). Thus, EGF-dependent phosphorylation of PG may modulate cell-cell adhesion not only by shifting PG’s own localization but also by disrupting the association with DSP and intermediate filaments. A phosphorylation-deficient PG mutant prevented the EGFR-dependent loss of DSP from junctions (Gaudry et al., 2001). Moreover, sustained tyrosine phosphorylation of PG, induced by pervanadate treatment of human keratinocytes decreased cell-cell adhesion as well as PG binding to E-cadherin and α-catenin (Hu et al., 2001). In support, EGFR inhibition blocked this phosphorylation and increased membrane-associated PG, which promoted cell-cell adhesion (Lorch et al., 2004; Bektas et al., 2013). In contrast to these data reporting a destabilization of desmosomes by EGFR signaling, Garrod et al. (2008) found that phosphorylated DSG2 and PG accumulated in pervanadate treated MDCK cells but this was accompanied by a stabilization of desmosomes and induction of hyperadhesion. Src kinase, which is activated by EGFR signaling, modified PG at Tyr643. This decreased the interaction of PG with proteins from AJ, such as E-cadherin and α-catenin and increased its interaction with DSP, thus promoting desmosome formation. In contrast, the tyrosine kinase Fer phosphorylated PG at Tyr549 and increased PG binding to α-catenin. These data suggest that tyrosine kinases like Src or Fer influence the association of PG with either AJs or desmosomes to regulate cell-cell adhesion and emphasize the importance of a careful analysis of the role of individual modifications (Miravet et al., 2003). In conclusion, PG’s function is regulated by phosphorylation downstream of the EGFR suggesting a role in dynamic remodeling of junctions but the role of individual tyrosine and serine/threonine phosphorylations and their interdependence is not yet fully understood.

Src kinase also mediated phosphorylation of PKP3 at Tyr195, which resulted in its release from desmosomes, suggesting that phospho-Tyr195 might play a role in desmosome disassembly. However, EGFR induced Tyr195 phosphorylation was transient and only detected when tyrosine phosphatases were inactivated (Neuber et al., 2015). In an attempt to identify peripheral desmosomal components that may modulate desmosome functions, Badu-Nkansah and Lechler detected several tyrosine phosphatases (tyrosine-protein phosphatase non-receptor type 11 and type 13) (Badu-Nkansah and Lechler, 2020). The presence of such phosphatases at desmosomes could explain the short half-life of PKP3 tyrosine phosphorylation under steady state conditions. EGFR signaling activates members of the cAMP-dependent, cGMP-dependent, and PKC (AGC) family kinases, that phosphorylate substrates at the AGC kinase consensus site RXXpS/T (R = arginine, X = any amino acid, S = serine, T = threonine). EGFR signaling induced PKP3 phosphorylation at this motif, affecting PKP3 localization (Muller et al., 2020). PKP3 phosphorylation was observed within a few minutes after EGF treatment which improved PKP3 association with lateral membranes thereby promoting desmosome assembly. Prolonged EGF treatment supported PKP3 sorting into tricellular contacts. Phosphorylation of PKP3 was mediated by the MEK/ERK pathway which activated the ribosomal S6 kinase family (RSKs). RSK1 and 2 directly phosphorylated PKP3 *in vitro* at Ser134/135 and their overexpression resulted in increased tricellular PKP3 localization. In contrast, RSK knockdown impaired PKP3 localization at tricellular contacts, which resulted in decreased cell-cell cohesion. Tricellular junctions are emerging as sites that integrate biochemical and mechanical signals to control local cell dynamics while maintaining tissue barrier function. Key functions include the regulation of cell division orientation, cytokinesis, planar cell polarity, collective cell migration, stem cell proliferation and cellular mechanical properties (Bosveld and Bellaiche, 2020). How PKP3 contributes to these functions remains to be elucidated.

Epidermal growth factor receptor signaling also modifies DSP and affects the associated keratin filaments. EGFR-mediated activation of ERK1/2 decreased DSP mRNA and protein amounts, whereas EGFR inhibition supported DSP recruitment to cell borders and increased DSP in the desmosome suggesting that EGFR-mediated transcriptional activation targets the *DSP* gene (Lorch et al., 2004; Pang et al., 2004). A direct phosphorylation of DSP by the EGFR/MAPK pathway has so far not been investigated although numerous phospho-tyrosine as well as phospho-threonine/serine residues have been detected in large scale screens (Moritz et al., 2010). However, glycogen synthase kinase 3 (GSK3) which is regulated by the PI3K pathway, phosphorylated six serine residues in the DSP C-terminus. Arginine methylation in the same region was required to recruit GSK3 to the DSP C-terminus suggesting that arginine methylation aids GSK3 kinase recognition to initiate DSP phosphorylation. This modulated DSP-keratin interactions and facilitated desmosome assembly. Overexpression of the DSP-R2834H mutant enhanced DSP-keratin associations and delayed junction assembly (Albrecht et al., 2015). Interestingly, these arginine methylation sites include Arg2834, which is mutated in patients suffering from arrhythmogenic cardiomyopathy (ACM).

Taken together, EGFR signaling regulates multiple desmosomal proteins by PTMs both on serine/threonine and on tyrosine residues. However, the effects of EGFR-mediated phosphorylation on the regulation of desmosomal adhesion are complex since activation of EGFR signaling can either stabilize or destabilize desmosomes. Further work is required to elucidate the subtleties of the mechanisms involved in these opposing responses. Because of the prevalent application of EGFR inhibitors in cancer treatment, it is important to understand their impact on epithelial regeneration and barrier formation in more detail.

#### Insulin and Insulin-Like Growth Factor 1 Signaling

**FIGURE 3:**
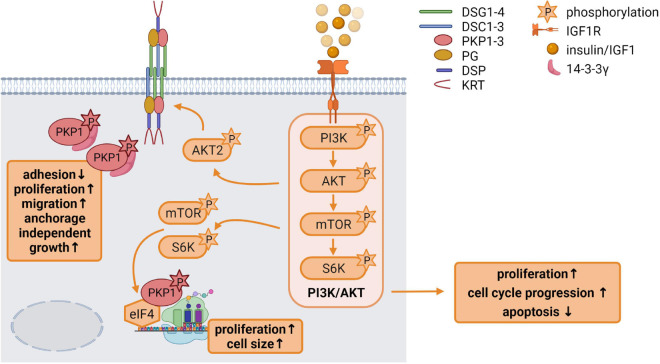
Insulin like growth factor 1 (IGF1) signaling is an important regulator of epidermal homeostasis *(created with biorender.com)*. Binding of IGF1 activates the IGF1R and the downstream PI3K/AKT signaling cascade *via* the phosphorylation of its components PI3K, AKT, mTOR, and S6K, resulting in increased proliferation and cell cycle progression as well as decreased apoptosis. Activated AKT2 phosphorylates PKP1, which translocates from the cell membrane to the cytoplasm. Phosphorylated cytoplasmic PKP1 is stabilized and protected from degradation *via* 14-3-3γ binding, resulting in impaired adhesion but increased proliferation, migration and anchorage independent growth. mTOR and S6K regulate binding of translation initiation factors of the eIF4 complex to mRNAs, thereby promoting protein biosynthesis. Phosphorylated PKP1 interacts with this translation initiation complex and stimulates eIF4A activity thereby facilitating unwinding of secondary structures in the 5′-UTR. The increase in protein biosynthesis correlates with increased proliferation and cell growth.

Insulin like growth factor 1 has a variety of functions in growth control and differentiation, cellular survival as well as tissue homeostasis. It is an important regulator of skin development and differentiation and IGF1R deficiency in keratinocytes disrupted epidermal homeostasis and stem cell maintenance (Muraguchi et al., 2019). In the skin, IGF1 is primarily derived from dermal cells. In general, binding of IGF1 to its receptor activates the receptor tyrosine kinase which initiates MAPK and particularly PI3K pathway activation (Figure 3; Hakuno and Takahashi, 2018).

As mentioned above, PKP1 can increase desmosome size by recruiting desmosomal proteins to the plasma membrane. This was counteracted by PKP1 phosphorylation that depended on IGF1 signaling through AKT2. AKT2, but not the related AKT1, phosphorylated PKP1, which enhanced and stabilized its cytoplasmic pool as indicated by a considerably increased half-life, whereas non-phosphorylated PKP1 was more rapidly degraded. This raises the question how phosphorylated PKP1 was protected from degradation in the cytoplasm. 14-3-3 proteins are a family of phospho-binding proteins that integrate and control multiple signaling pathways. Phosphorylation of target proteins occurs frequently in intrinsically disordered regions, such as the PKP1 N-terminal domain and often occur in pairs with each phosphorylation interacting with a phospho-binding pocket of a 14-3-3 dimer. 14-3-3 docking to the phosphorylated target proteins can have wide ranging effects. For instance, 14-3-3 binding can modulate intracellular localization, complex formation, conformation and protein stability (Pennington et al., 2018). The 14-3-3γ isoform associated specifically with PKP1 phosphorylated by AKT2 at Ser155 to protect it from degradation (Rietscher et al., 2018) (summarized in Figure 3). While phosphorylation of PKP1 promoted complex formation with 14-3-3γ and eIF4A1, DSP and DSG1 interactions were considerably reduced in a phospho-mimetic PKP1 mutant. Thus, PKP1 phosphorylation by IGF1/AKT2 weakened its desmosome association resulting in the translocation of PKP1 to the cytoplasm. This correlated with reduced intercellular adhesion and an increased activity of PKP1 in the stimulation of translation accompanied by an increase in proliferation (Wolf et al., 2013). Thus, cytoplasmic PKP1 may contribute to the maintenance of a proliferating cell pool and facilitate cell dynamics in the basal epidermal keratinocytes while dephosphorylated PKP1 promotes desmosome formation and stability in the suprabasal keratinocytes.

#### Hippo Signaling

**FIGURE 4:**
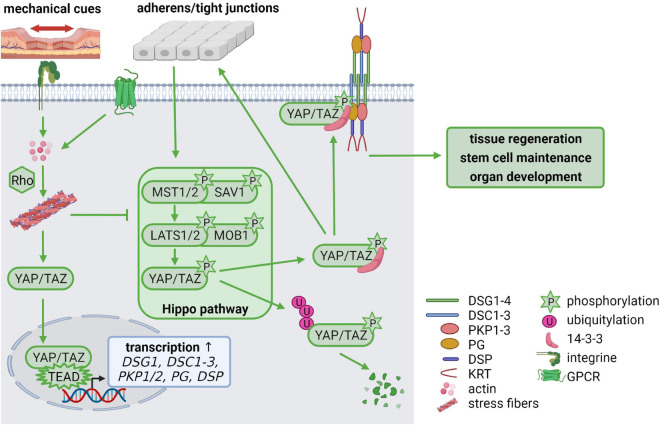
Mechanical cues regulate cellular homeostasis *via* Hippo signaling *(created with biorender.com)*. Cell-cell contacts control the activation of the Hippo signaling cascade *via* phosphorylation of MST(Hippo)/SAV and LATS/MOB. The phosphorylated downstream targets YAP/TAZ are degraded *via* ubiquitylation or stabilized in the cytoplasm by 14-3-3-binding, which facilitates YAP/TAZ association with cell-cell contacts including adherens junctions, tight junctions and desmosomes. Mechanical tension activates RhoA *via* integrin signaling which promotes stress fiber formation. G protein-coupled receptors (GPCRs) can also activate Rho to promote stress fiber assembly. This inhibits LATS leaving YAP/TAZ in an unphosphorylated state. Unphosphorylated YAP/TAZ translocates from the cytoplasm to the nucleus, where it forms a complex with TEAD transcription factors, resulting in increased transcription of TEAD target genes including *DSG1*, *DSC1-3*, *PKP1/2*, *PG*, and *DSP*. Thus, mechanical cues control desmosomal gene expression *via* the Hippo cascade but, in a feedback mechanism, desmosomes modulate mechanosignaling by capturing YAP/TAZ at the plasma membrane, to maintain the balance between proliferation, differentiation, migration, and invasion.

During the last few years, it has become increasingly clear that not only growth factor signaling is essential for cellular homeostasis but that mechanical stimuli are equally important. The Hippo pathway is regulated by mechanical stimuli and enables cells to adapt to changes in their environment, thereby regulating tissue regeneration, stem cell maintenance, organ development and carcinogenesis (Rausch and Hansen, 2020). Several Hippo pathway components temporally localize to junctional complexes where the upstream Hippo pathway components are activated. Intrinsic and extrinsic signals, such as cell-cell contacts, stiffness of the extracellular matrix and mechanical force activate the signaling cascade by phosphorylation of the scaffold protein Salvador (SAV) and the mammalian STE20-like kinase 1/2 (MST1/2, Hippo in Drosophila) as well as the Mps one binder kinase activator-like 1 (MOB1) and the large tumor suppressor 1/2 (LATS1/2), which in turn phosphorylate the downstream targets Yes-associated protein (YAP) and the transcriptional co-activator with PDZ-binding motif (TAZ). Phosphorylation inactivates YAP and TAZ, resulting in their cytoplasmic retention by 14-3-3 proteins where they are either proteasomally degraded or captured at the cell membrane. When Hippo signaling is inactive, unphosphorylated YAP/TAZ enters the nucleus, forms complexes with TEAD1-4 transcription factors and promotes expression of their transcriptional targets (Figure 4; Rausch and Hansen, 2020).

Most studies deal with the role of AJs, tight junctions and focal adhesions in recruiting Hippo components to cell contacts to regulate Hippo signaling (reviewed in Boopathy and Hong, 2019; Dasgupta and McCollum, 2019). In contrast, only few studies investigated the regulation of YAP activity by desmosomal proteins or desmosomes as targets of Hippo signaling (Chen et al., 2014; Uttagomol et al., 2019). Chen et al. (2014) reported that the desmosomal plaque proteins PKP2, DSP, and PG affect the activation and localization of Hippo pathway components in cardiomyocytes. PKP2 knockdown enhanced the activation of neurofibromin 2 (NF2 or Merlin), which in turn phosphorylated and activated MST1/2 and LATS1/2 kinases. Consequently, phosphorylation and inactivation of YAP prevented TEAD activation and reduced TEAD target gene expression. YAP activation was rescued by double knockdown of PKP2 and LATS1/2 indicating that PKP2 was required to limit LATS1/2 activity in these cells. Similarly, DSP- and PG-deficient mouse models of ACM showed increased levels of active NF2, resulting in an increase in phosphorylated MST1/2 and YAP, which predominantly localized at the cell membrane. YAP and PG were co-immunoprecipitated, suggesting that this interaction might contribute to the localization of Hippo pathway components at cell junctions (Chen et al., 2014). Thus, PKP2, DSP, and PG can alleviate YAP signaling to allow target gene expression and facilitate proliferation and regeneration.

Stretching of HaCaT and oral-mucosa derived keratinocytes resulted in elevated expression of desmosomal proteins, indicating that desmosomes respond to mechanical load. Stretched keratinocytes revealed nuclear localization of YAP, whereas phospho-YAP localized at cell borders. DSG3 depletion correlated with reduced expression of YAP and phospho-YAP suggesting that DSG3 stabilizes phosphorylated YAP. Capturing phospho-YAP by DSG3 depended on PKP1 and 14-3-3 binding to YAP (Figure 4). Stretching of DSG3-depleted keratinocytes enhanced the nuclear localization of both, YAP and phospho-YAP, indicating a role of DSG3 in the cytoplasmic retention of phospho-YAP under mechanical strain (Uttagomol et al., 2019).

The actin cytoskeleton is known to be highly responsive to mechanical stresses and plays an important role in the regulation of YAP/TAZ by mechanical cues. In general, increasing F-actin levels promotes YAP/TAZ nuclear localization, whereas loss of F-actin causes YAP/TAZ accumulation in the cytoplasm (Dasgupta and McCollum, 2019). Rho GTPases regulate actin organization and several studies have indicated that Rho GTPases are essential mediators connecting mechanical stimuli and the actin-dependent Hippo-YAP regulation (Figure 4). Rho stimulates the assembly of contractile actin stress fibers by activating Rho-associated kinase (ROCK) and mDia1/2, while Rac and Cdc42 promote lamellipodia and filopodia formation. Activated Rho strongly enhanced YAP/TAZ activity and treatment of cells with a ROCK inhibitor reduced nuclear YAP/TAZ (Seo and Kim, 2018). Intriguingly, although not directly linked to the actin cytoskeleton, desmosomes can also influence actin organization (Hatzfeld et al., 2017). Loss of PKPs from human or mouse keratinocytes resulted in changes in cortical actin organization (Godsel et al., 2010; Keil et al., 2016). Overexpression of a PKP1 mutant, that lacked its desmosome binding domain, induced filopodia and long cellular protrusions, where PKP1 colocalized with actin filaments suggesting a role of PKP1 in regulating actin cytoskeleton dynamics (Hatzfeld et al., 2000). However, it is not clear whether PKPs regulate RhoA activity and stress fiber formation directly or indirectly, by influencing the localization or activity of a Rho guanine exchange factor (GEF) or a Rho GTPase activating protein (GAP). The question if desmosome-dependent remodeling of the actin cytoskeleton affects Hippo signaling has so far not been addressed.

As mentioned before, DSG1, DSC1-3, DSP, PKP1, PKP2, and PG were identified as putative TEAD4 targets (Liu et al., 2016) suggesting a feedback mechanism where inactive Hippo signaling promotes TEAD target gene expression including desmosomal genes thereby promoting desmosome formation (Figure 4). When desmosome formation reaches a threshold, YAP would be captured at desmosomes to prevent its nuclear localization and to limit target gene expression. This model supports the hypothesis that desmosomal proteins play an important role in regulating Hippo signaling, thereby affecting proliferation, differentiation, migration and invasion.

#### Wnt Signaling

Wnt signaling is another indispensable regulator of skin development and regeneration. Wnt pathways can be divided into β-catenin-dependent (canonical) and β-catenin-independent (non-canonical) Wnt signaling. Very briefly, in the absence of Wnt, cytoplasmic β-catenin is phosphorylated and becomes degraded by a destruction complex, composed of the core proteins Axin, casein kinase 1α (CK1α), adenomatous polyposis coli (APC), and GSK3β. Upon binding of Wnt ligands to the frizzled (FZ) receptor and coreceptor low-density lipoprotein receptor-related protein 5/6 (LRP5/6), disheveled (DVL) is recruited for the inhibition of the destruction complex. Stabilized cytoplasmic β-catenin enters the nucleus to act as a transcriptional co-activator for T-cell factor/lymphoid enhancer binding factor (TCF/LEF) and activates the transcription of Wnt-responsive genes. In the β-catenin-independent non-canonical Wnt pathways, binding of Wnt isoforms to either FZ or tyrosine kinase-like receptors, can trigger multiple signaling cascades, including activation of calmodulin-dependent protein kinase II (CaMKII), PKC or the small Rho GTPases Rho, Rac, and Cdc42. Wnt-dependent signaling is required for differentiation of ectodermal cells into the epidermal fate and plays a crucial role in the maintenance, activation, and fate determination of the skin stem cell populations (Veltri et al., 2018). Besides β-catenin, PG also participates in Wnt signaling by competing with β-catenin for degradation and transcriptional activation of TCF/LEF (Huber and Petersen, 2015; Aktary et al., 2017). Moreover, several other desmosomal proteins, e.g., DSG2, DSC3, PKP1-3, and DSP directly or indirectly affected Wnt signaling (Hardman et al., 2005; Yang et al., 2012; Miyazaki et al., 2016; Calore et al., 2019; Khudiakov et al., 2020; Hong et al., 2021). Wnt pathway components have been described to modulate stability, localization and/or function of desmosomal proteins. Although the particular PTMs have not been characterized, the amount of PG and its localization was influenced by exogenous Wnt-1 expression (Bradley et al., 1993; Papkoff et al., 1996). Like PG, PKP1 translocated to the nucleus upon stimulation by Wnt3a and LiCl, suggesting Wnt-dependent PTMs (Miyazaki et al., 2016). PKP3 associated with components of the β-catenin destruction complex, such as GSK3β and Axin and was degraded upon their overexpression. Moreover, PKP3 was stabilized in the presence of a Wnt ligand, translocated into the nucleus and stimulated Wnt reporter gene expression (Hong et al., 2021). Thus, PKP3 localization and amount can be regulated through Wnt-dependent PTMs. If and how PKP3 affects Wnt-dependent gene expression needs to be elucidated. Furthermore, GS3K which can be activated by Wnt as well as PI3K/AKT signaling, phosphorylated the DSP tail domain, thereby modulating DSP-keratin complexes and thus desmosome assembly (Albrecht et al., 2015). Although various desmosomal proteins are apparently effectors as well as regulators of Wnt signaling, the complex mechanistic interrelations are only beginning to emerge.

## Desmosomal Proteins as Effectors: Control of Proliferation

The regulation of proliferation might be an essential function of desmosomal proteins. Genodermatoses caused by mutations of desmosomal proteins are often accompanied by dysregulated proliferation of keratinocytes (reviewed in Najor, 2018; Lee and McGrath, 2021). This phenotype is also obvious in various animal models that analyzed the knockout or the misexpression of certain desmosomal proteins (Supplementary Table 1). Moreover, desmosomes can adapt their adhesive properties in response to tissue wounding to facilitate wound healing (Wallis et al., 2000; Thomason et al., 2012). Therefore, it is tempting to speculate that desmosomal proteins participate in a phenomenon known as contact inhibition of proliferation (CIP). CIP is a fundamental property which enables normal cells to arrest cell proliferation and initiate differentiation when they contact each other and is observed in most epithelial cells. CIP is reversed in physiological conditions requiring rapid cell growth and proliferation, such as wound healing and tissue regeneration. Loss of contact inhibition leads to uncontrolled cell growth and malignant transformation, resulting in tumor formation indicating that the balance between proliferation and adhesion is crucial for maintaining epithelial integrity. Tissue regeneration and wound repair are ensured by stem cells, located within specialized niches e.g., in the interfollicular epidermis or in the intestinal crypts. Tissue homeostasis requires that the number of cells lost is compensated by cell divisions providing the same number of cells. Whereas the role of AJs and E-cadherin in CIP has been extensively studied, the role of desmosomal proteins in controlling proliferation vs. differentiation is only beginning to emerge (Gonzales and Fuchs, 2017; Mendonsa et al., 2018). A direct involvement of desmosomal proteins in cell cycle regulation has so far not been reported. However, they have been implicated in the regulation of upstream mitogenic and Hippo signaling pathways as well as translational control suggesting an indirect role in the control of the cell cycle and proliferation.

### Control of Mitogenic Signaling by Desmosomal Proteins

Numerous studies indicate the differential involvement of desmosomal cadherins in the control of proliferation *via* EGFR signaling (see Figure 2). DSG1 whose expression is restricted to suprabasal keratinocytes, is not only required for maintaining epidermal integrity but also supports keratinocyte differentiation in a desmosome-independent manner. DSG1 facilitated keratinocyte progression to terminal differentiation by suppressing EGFR signaling (Getsios et al., 2009). This was mediated by a DSG1/Erbin/SHOC2 complex, which prevented SHOC2-dependent association of ERK with its activators RAS/RAF, thus attenuating ERK activity and driving differentiation (Harmon et al., 2013). Desmosomes also act as a scaffold to place the constitutive photomorphogenesis 9 (COP9) signalosome close to the EGFR. DSG1 and DSP interacted with the COPS3 subunit of the COP9 signalosome. Since loss of COPS3 as well as DSG1 increased EGFR phosphorylation and compromised keratinocyte differentiation, the authors suggested that DSG1 inhibited EGFR signaling and promoted differentiation in a COP9 signalosome-dependent manner. The molecular mechanism comprised de-neddylation of the EGFR by the COP9 signalosome which triggered ubiquitination and EGFR removal from the cell surface and finally its degradation. This dampened EGFR signaling and consequently cell division, and allowed differentiation to proceed. These data support a model where DSG1-dependent scaffolding of the COP9 signalosome facilitates epidermal differentiation by controlling EGFR dynamics (Najor et al., 2017).

While DSG1 promoted differentiation, the general knockout of DSG2 was associated with embryonic lethality short after implantation, and decreased embryonic stem cell proliferation (Eshkind et al., 2002) suggesting a positive role in the regulation of proliferation. In pluripotent stem cells, DSG2 was essential for self-renewal and suppression of differentiation (Park et al., 2018). Overexpression of DSG2 in basal keratinocytes under the control of the keratin (KRT) 14 promoter did not affect proliferation in general but promoted wound healing associated with elevated EGFR/MAPK activity (Cooper et al., 2018). However, ectopic expression of DSG2 in suprabasal keratinocytes under the control of the involucrin (IVL) promoter activated EGFR signaling and downstream pathways, converging in elevated proliferation and epidermal hyperplasia (Brennan et al., 2007). Thus, ectopic expression of DSG2, which is normally restricted to basal keratinocytes, was sufficient to increase proliferation in suprabasal cells. In agreement with a positive role in growth control, elevated DSG2 levels were observed in several cancers where DSG2 promoted proliferation (Cai et al., 2017; Han et al., 2018; Qin et al., 2020; Sun et al., 2020). Loss of DSG2 suppressed colon cancer cell proliferation through inhibition of EGFR signaling (Kamekura et al., 2014). DSG2 was not only overexpressed and colocalized with EGFR in cutaneous SCCs *in vivo*, but also promoted Src-mediated EGFR activation required for proliferation and migration in HaCaT and A431 cells (Overmiller et al., 2016). Such an extradesmosomal function of DSG2 in regulating proliferation and migration through activation of EGFR/MAPK pathway was confirmed in cervical cancer and lung adenocarcinoma cell lines (Jin et al., 2020; Zhou and Li, 2020).

DSG3 is most abundant in basal proliferating keratinocytes. Its ectopic expression in suprabasal keratinocytes under the control of the KRT1 promoter led to hyperproliferation and interfered with epidermal differentiation. Cells expressing the proliferation marker Ki-67 were not restricted to the basal layer as in wild type skin but also found in the suprabasal layer (Merritt et al., 2002). A transgenic mouse expressing N-terminally truncated DSG3 revealed dramatically reduced numbers of smaller and structurally altered desmosomes. Disruption of desmosomes was especially prominent in the paws and tail. A marked increase in cell proliferation was elicited in areas where cell adhesion was not completely lost (Allen et al., 1996). In HaCaT and MDCK cells, a DSG3 knockdown resulted in impaired desmosome assembly and defects in cell adhesion as well as reduced proliferation with a reduction in G1/S phase transition and reduced colony size. In contrast, overexpression of DSG3 promoted cell proliferation (Mannan et al., 2011). In agreement, DSG3 was highly expressed in head and neck cancer and its expression correlated with proliferative and invasive properties of these cancer cell lines (Chen et al., 2007). Mechanistically, DSG3 silencing induced changes in desmosome composition with a loss of PG from the cell membrane and its translocation to the nucleus. This promoted an interaction of PG with the transcription factor TCF. Since PG is a negative regulator of TCF, nuclear PG alleviated TCF’s transcriptional activity and as a consequence, expression of c-MYC and cyclin D1 leading to a cell cycle arrest at the G0/G1 phase (Chen et al., 2013). Since the knockdown DSG3 reduced the expression and activation of EGFR (Ri et al., 2019), DSG3 might also regulate proliferation through EGFR signaling. Moreover, a crosstalk between DSG3 and EGFR signaling has been suggested in several reports dealing with PV pathogenesis. However, DSG3-mediated control of Hippo signaling by sequestration of YAP may also contribute to DSG3-dependent control of keratinocyte proliferation (Uttagomol et al., 2019) suggesting that DSG3 may contribute to coordinate cell signaling pathways to control CIP.

Mice lacking DSC1 show epidermal fragility accompanied by barrier defects and abnormal differentiation as well as epidermal thickening and hyperproliferation. As in DSG3-overexpressing skin, proliferating cells were not restricted to the basal layer, but also detected in suprabasal cells suggesting a role of DSC1 in suppressing proliferation by a so far unknown mechanism (Chidgey et al., 2001). However, the ectopic expression of DSC1 in basal keratinocytes under the control of the KRT14 promoter revealed no changes in keratinocyte proliferation, stratification, or differentiation (Henkler et al., 2001).

The general knockout of DSC2 has no obvious phenotype, suggesting compensatory mechanism of other desmosomal cadherins *in vivo* (Rimpler, 2014). However, in enterocytes DSC2 knockdown increased proliferation as indicated by elevated numbers of cells in S phase and activation of EGFR/AKT/β-catenin signaling (Kolegraff et al., 2011). A similar observation was made in prostate cancer cells, where a DSC2 knockdown led to enhanced expression of the cell cycle regulators cyclin D1, CDK2, cyclin B1, and CDK1 and promoted proliferation whereas overexpression of DSC2 led to downregulation of the same genes (Jiang and Wu, 2020). Taken together, these results suggest a role of DSC2 in suppressing proliferation in agreement with a role as a tumor suppressor.

A DSC3 knockout revealed severe epidermal hyperplasia in adult mice due to increased basal cell proliferation and reduced cell adhesion with skin blistering and hair loss but did not affect desmosome size (Chen et al., 2008). In agreement with a proliferation suppressive function, DSC3 downregulation by promoter methylation was reported in lung cancer (Cui et al., 2012) and prostate cancer, where DSC3 depletion correlated with poor prognosis (Pan et al., 2014). Cui et al. (2012) reported that DSC3 decreases EGFR/RAS/RAF/MAPK signaling in human lung cancer cells. High expression of DSC3 resulted in reduced phosphorylation of ERK1/2 and G0/G1 cell cycle arrest which blocked proliferation, whereas knockdown of DSC3 increased the amount of phospho-ERK1/2 (Cui et al., 2012). A negative correlation between DSC3 expression, PI3K/AKT signaling and proliferation was also found in colorectal cancer (Cui et al., 2019). However, conflicting results have been reported concerning DSC3’s role in cancer where Dsc3 either suppressed or facilitated proliferation, depending on tumor or cell type. For example, DSC3 was highly expressed in ovarian cancer cells, and promoted proliferation by a regulatory loop of DSC3, EGFR and PI3K/AKT signaling through follicle stimulating hormone (Yang X. et al., 2015).

Taken together, desmosomal cadherins appear to be critical regulators of context dependent proliferation control. Available data on the molecular mechanisms suggest that many of the effects converge on EGFR/MEK/ERK and PI3K/AKT-mediated signaling (summarized in Figure 2).

The targeted deletion of PG in basal keratinocytes promoted their proliferation (Li et al., 2012). Since PG is regulated through EGFR signaling and can suppress p38MAPK activation, PG may modulate EGFR-dependent control of proliferation (Spindler et al., 2014). PG has been shown to control the transcription of proliferation-promoting genes. Although skeletal muscle lacks “classic” desmosomes, they express several desmosomal proteins. In normal muscle, PG associated with the insulin receptor and the p85 subunit of PI3K to promote PI3K-AKT-Forkhead box O1 (FOXO1) signaling required for muscle cell growth and survival (Cohen et al., 2014). Moreover, PG silencing reduced the expression of AKT and attenuated insulin signaling including insulin-induced glucose uptake in adipocytes (Negoita et al., 2020). Whether PG is involved in regulating insulin sensitivity in epithelial cells remains to be determined.

PKP2 is associated with proliferation control through EGFR signaling: PKP2 interacted with the EGFR *via* its N-terminal domain and enhanced EGF-dependent and EGF-independent EGFR dimerization and phosphorylation (Figure 2). In support, PKP2 knockdown reduced EGFR phosphorylation and attenuated EGFR-mediated signal activation, resulting in a significant decrease in proliferation and migration of breast cancer cells (Arimoto et al., 2014). In lung adenocarcinoma, PKP2 knockdown suppressed proliferation as indicated by reduced numbers of cells in S phase (Wu et al., 2021) whereas PKP2 overexpression led to enhanced proliferation and colony formation (Hao et al., 2019). PKP2 is mainly expressed in cardiomyocytes and heterozygous mutations in the PKP2 gene are a common cause of ACM (Gerull et al., 2004). Therefore, many studies have focused on its role in cardiomyocytes and have detected a link between PKP2 and proliferation control. PKP2 knockdown in HL-1 cardiomyocytes suppressed E2F1 transcription required for G1/S phase progression and proliferation (Gurha et al., 2016). In contrast to these reports pointing to a proliferation promoting function of PKP2, Matthes et al. (2011) reported enhanced Bromodeoxyuridine (BrdU) incorporation in response to PKP2 depletion in explants from neonatal rat hearts, indicative of a proliferation suppressive function of PKP2. So far, it is not known if these contradictory findings can be explained by distinct signaling pathway activation in the various model systems which may result in differential PTMs of PKP2. These could switch PKP2 dependent functions in a similar way as described for PKP1 as a function of IGF1 signaling.

The contribution of all three PKPs to cancer appears to be context dependent and a result of their multiple functions in cell adhesion and signaling (Hatzfeld et al., 2014). Breuninger et al. (2010) studied the role of PKPs in prostate cancer cells. PKP3 expression was enhanced whereas PKP1 and PKP2 were reduced or unaffected, respectively. Overexpressed PKP3 localized with other desmosomal proteins at cell membranes but in addition in the cytoplasm and enhanced BrdU incorporation, which suggested a pro-proliferative role of PKP3 (Breuninger et al., 2010). High PKP3 expression was also observed in non-small cell lung carcinoma, which correlated with poor prognosis and survival. PKP3 knockdown in these cancer cells led to impaired growth, whereas overexpression promoted cell growth (Furukawa et al., 2005). The molecular mechanism how PKP3 modulates proliferation is so far not understood. An attractive possibility could be that PKP3 besides being regulated itself by RSK downstream of EGFR signaling, modulates EGFR pathway activity in a feedback loop.

Evidence from diseases caused by DSP haploinsufficiency suggested that alterations in DSP expression caused disruption of tissue structure but in addition changes in keratinocyte proliferation (Najor, 2018; Lee and McGrath, 2021). During embryogenesis, DSP is required to establish polarity and assemble desmosomes. DSP knockout embryos did not survive beyond E6.5, owing to a loss or instability of desmosomes and tissue integrity. Moreover, embryos were significantly smaller than normal and proliferation was considerably reduced as judged by decreased BrdU incorporation (Gallicano et al., 1998). However, it is not clear to what extent the reduced proliferation was directly linked to DSP loss or if the gross perturbation of tissue integrity indirectly impeded proliferation. In human HaCaT keratinocytes, DSP was shown to regulate cell cycle progression and proliferation. DSP knockdown not only disturbed desmosome number as indicated by reduced levels of all major desmosomal proteins, but also increased BrdU incorporation, indicating an increase in cells in S phase. Proliferative changes were associated with elevated activation of ERK1/2 and AKT which was sustained when cells reached confluence, whereas control cells downregulated ERK activity upon confluence suggesting a role of DSP in CIP (Wan et al., 2007). In cardiac but not in epidermal cells, DSP loss elevated the activity of K-RAS, an upstream activator of ERK1/2, confirming a role of DSP in suppressing mitogenic signaling (Kam et al., 2018). Schmitt-Graeff et al. (2007) reported downregulation of DSP and PKP1 during progression to SCC. Proliferative activity was inversely correlated with desmosomal protein expression in patient samples from SCC, which is compatible with an anti-proliferative and tumor suppressive role for DSP (Schmitt-Graeff et al., 2007). Many other studies did not detect a direct role of DSP in regulating proliferation. For example, a DSP knockout in human keratinocytes led to a loss of desmosomes with impaired cellular adhesion but did not affect proliferation (Wanuske et al., 2021). In an intestine specific DSP knockout, proliferation was also not affected. Surprisingly, cellular adhesion was maintained and keratin localization was unaltered in this tissue, although the intermediate filaments were not anchored at desmosomes any more (Sumigray and Lechler, 2012). Thus, the role of DSP in proliferation requires further studies to elucidate the context that enables a growth-suppressive function of DSP *via* ERK signaling. Although DSP reveals a substantial extradesmosomal pool, the function of this pool remains essentially elusive. In an attempt to identify DSP interactions that might modulate canonical or non-canonical desmosome functions, a targeted proximity labeling assay was performed in epidermal keratinocytes. Quantitative mass spectrometry analysis identified a diverse array of new interactions with broad molecular functions including transcription factors and transcriptional coactivators (including YAP), translation initiation factors and many regulatory proteins (Badu-Nkansah and Lechler, 2020). Interestingly, numerous SH2/SH3 adapter proteins as well as protein tyrosine phosphatases have also been identified, further supporting the assumption of a close connection between desmosomes and growth factor signaling. Elucidating the role of such interactors will substantially advance our understanding of context dependent DSP functions.

### Control of Protein Synthesis by Desmosomal Proteins

The overall rate of protein synthesis has to keep pace with the proliferation rate to maintain cell size and functionality (Miettinen et al., 2019). Therefore, cell proliferation strongly depends on the synthesis of new proteins (Pardee, 1989; Polymenis and Aramayo, 2015). This is supported by reports showing that modifications of the translation machinery can affect cell proliferation rates and that deregulation of protein synthesis can be a driver of cell transformation (Silvera et al., 2010; Truitt and Ruggero, 2016). mRNA translation is mostly controlled at the level of initiation during which the small 40S ribosomal subunit is recruited to the 5′-cap structure of the mRNA and scans the mRNA 5′-UTR for the start codon. Following recognition, the 80S initiation complex is assembled at the start codon and elongation will proceed. Translation initiation requires several eukaryotic translation initiation factors (eIFs) and is partly regulated by the mammalian target of rapamycin (mTOR) signaling pathway which senses and responds to nutrient availability, energy sufficiency, stress, hormones and mitogens to modulate protein synthesis (Ma and Blenis, 2009). mTOR signaling *via* ribosomal S6 kinases (S6Ks) regulates eIF4E binding to the mRNA cap and recruitment of eIF4A, eIF4B, and eIF4G. eIF4A is an RNA helicase that is capable of unwinding mRNA secondary structures facilitating the translation of mRNA species containing inhibitory secondary structures in their 5′ untranslated region. PKP1 was identified as a component of the cap-binding translation initiation complex where it associated directly with eIF4A1. PKP1 not only stimulated the recruitment of eIF4A1 into the cap complex but also promoted its helicase activity. The stimulation of translation upon PKP1 overexpression correlated with an upregulation of proliferation and cell size (Figure 3; Wolf and Hatzfeld, 2010; Wolf et al., 2010).

The dual function of PKP1 in increasing desmosome size and adhesion on the one hand and in stimulating translation and proliferation on the other hand pointed to a role of this protein in mediating CIP. Obviously, PKP1’s role depended on its localization which was regulated by the IGF1/AKT2 signaling axis, a pathway implicated in the general regulation of translation. Unregulated activation of AKT2 was observed in papillomas and in human papilloma virus (HPV) induced epidermal tumors and was characteristic of SCC (O’Shaughnessy et al., 2007). Moreover, AKT2 was upregulated by ultraviolet (UV) radiation, the most important skin carcinogen (Sully et al., 2013). These data place PKP1 among the effectors of AKT2 signaling and suggest a role of PKP1 in the uncontrolled proliferation of certain skin carcinoma. In agreement, Wolf et al. (2013) showed that a PKP1 mutant that mimics AKT2 induced phosphorylation confers anchorage independent growth.

### Control of Gene Expression by Desmosomal Proteins

Gene expression is primarily controlled at the transcriptional level. Li et al. (2012) reported epidermal thickening, impaired inflammation responses, and disrupted desmosome assembly in epidermis specific PG knockout mice. Proliferation was increased as shown by elevated BrdU incorporation, with proliferating cells restricted to the basal layer (Li et al., 2012). In MCF-7 breast cancer cells, overexpression of PG suppressed proliferation, whereas its knockdown promoted proliferation. This correlated with increased levels of tumor promoters such as ERBB2 and Snail and decreased levels of tumor suppressors. Transcriptional activity of the tumor suppressor p53 was enhanced in the presence of PG suggesting that PG regulates gene expression in conjunction with p53 (Aktary et al., 2013, 2017). PG was also reported to repress expression of the c-MYC proto oncogene in a LEF-1 dependent way suggesting that PG blocked LEF-1 transcriptional activity. Since PG-mediated suppression of MYC was similar in both wild type and β-catenin-null keratinocytes, this effect did not depend on a competition between PG and β-catenin for LEF-1. Moreover, ChIP experiments with PG antibodies demonstrated an association of PG and LEF-1 with the MYC promoter in keratinocytes undergoing growth arrest, supporting a role of PG in transcriptional regulation (Williamson et al., 2006). PG may also function to potentiate death in cells damaged by apoptotic stimuli, perhaps limiting the potential for the propagation of mutations and cellular transformation. Since PG knockout keratinocytes showed increased levels of anti-apoptotic B-cell lymphoma extra-large (BCL2L1 alias BCL-XL), the resulting protection from apoptosis might also be mediated by the regulation of transcription (Dusek et al., 2007).

In *Xenopus* embryos, PKP3 associated with the transcription factor erythroblast transformation specific variant 1 (ETV1) and positively modulated ETV1-dependent transcriptional activation. Since ETV1 promotes metastasis of prostate cancer, one might speculate that elevated expression of PKP3 stimulates ETV1 target gene expression to promote proliferation and metastasis in prostate carcinoma (Munoz et al., 2014).

In conclusion, desmosomal proteins regulate proliferation in an adhesion-dependent and adhesion-independent manner. Desmosomal dysfunction can promote cancer development, which is accompanied by enhanced cell cycle progression, resulting in hyperproliferation and tumor growth. Some desmosomal proteins, such as DSG2, DSG3, and PKP2, are highly expressed in many cancers and promote proliferation *via* EGFR signaling. This promotes cell cycle entry and progression by increasing the expression of proliferation targets, such as cyclin D1, cyclin A2, and c-MYC. In contrast, DSCs seem to suppress proliferation. The role of PKP3 and DSP in regulating proliferation requires further investigation. Contradictory results on the correlation between expression and proliferation rates may be explained by different tumor entities and cell lines with considerable differences in signaling pathway activation. It is therefore necessary to fully understand the functional relationship between signaling pathway components and their desmosomal targets and how these signals control non-desmosomal functions.

## Desmosomal Proteins as Effectors: Regulation of Inflammation

Epithelial cells not only play an important role in maintaining the physical barrier between the host and the environment, but also participate in immune responses. Disruption of the barrier induces an innate immune response. Such inflammatory processes must ensure a rapid and efficient host defense in response to pathogens, toxic compounds or endogenous harmful signals, and to initiate wound healing. At the same time, excessive and/or persistent inflammation may lead to septic shock, induction of autoimmunity, non-healing chronic wounds, increased fibrosis or cancer. The initial insults are sensed through several families of pattern recognition receptors (PRRs), including Toll-like receptors (TLRs). These PRRs are expressed on myeloid as well as on epithelial cells, including intestinal epithelial cells (IECs) and keratinocytes. In a very simplified view, upon recognition of extrinsic pathogen associated or intrinsic danger associated molecular patterns (PAMPs and DAMPs), PRRs trigger signaling cascades that lead to the nuclear translocation and activation of transcription factors like NFκB, AP-1 and interferon regulatory factors (IRFs), resulting in the transcription of numerous genes essential to modulate immune responses. However, the detailed molecular mechanisms that characterize epithelial-specific inflammatory responses are only partially understood (Pasparakis et al., 2014; Richmond and Harris, 2014; Piipponen et al., 2020a). Here we discuss how desmosomal proteins might contribute to the regulation of inflammation beyond ensuring the physical barrier of epithelia.

It is known that desmosomal proteins react to pro-inflammatory cytokines as well as inflammatory triggers. However, it is unknown if this is a consequence of inflammation or rather part of a regulatory mechanism to keep inflammatory responses in shape. Pro-inflammatory cytokines, such as tumor necrosis factor α (TNF-α), interleukin-1β (IL-1 β), and interferon-γ (IFN-γ) released during mucosal inflammation induced intracellular DSG2 cleavage and ectodomain shedding, which compromised intercellular adhesion, promoted proliferation through ERBB2/3 and MAPK pathways and induced apoptosis (Kamekura et al., 2015; Yulis et al., 2018). UV irradiation, which provokes TLR3-dependent inflammation (Bernard et al., 2012), and polyinosinic/polycytidylic acid (poly I:C) mediated activation of TLR3 altered desmosomal protein and transcript amounts in keratinocytes (Bayerl et al., 1995; Li et al., 2001; Murakami et al., 2001; Sesto et al., 2002; Rundhaug et al., 2005; Borkowski et al., 2013) and resulted in their redistribution from cell borders into the cytoplasm (Dusek et al., 2006). DSG1 and DSC1 levels were reduced by UV-B exposure of keratinocytes accompanied by differentiation defects. Intriguingly, ectopic expression of DSG1 prevented UV-B induced differentiation defects, suggesting that DSG1 contributes to UV-triggered inflammatory responses (Johnson et al., 2014). Several reports describe a change in desmosomal cohesion from a hyperadhesive to a more dynamic calcium-dependent state at the wound edge at least partially regulated through PKCα (reviewed in Garrod, 2010; Garrod and Tabernero, 2014). Tissue wounding requires innate and adaptive immune responses to restore tissue integrity (Piipponen et al., 2020a). Since PKCα can be activated through TLR3 signaling (Johnson et al., 2007), it is tempting to speculate that TLR signaling regulates desmosomal cohesion during wound induced inflammation.

Local and/or systemic inflammation as well as recurrent infections frequently accompany diseases caused by dysfunctional desmosomal proteins (reviewed in Broussard et al., 2015; Najor, 2018; Lee and McGrath, 2021). Flawed inflammatory responses have also been reported from animal models for desmosomal genes (Supplementary Table 1). This raises the question if desmosomal proteins fulfill an active role in the regulation of inflammatory processes besides being targeted by inflammatory responses. Although, the defective physical barrier is often blamed to trigger these inflammatory phenotypes, it is obvious that desmosomal proteins participate in the regulation of EGFR/MAPK, Wnt, PI3K/AKT, and Hippo signaling. These pathways intensively crosstalk with pro- and anti-inflammatory pathways (Yeung et al., 2018; Jridi et al., 2020), suggesting that desmosomal proteins may directly modulate inflammation. In addition, several studies using animal as well as cell culture models described a direct connection between desmosomal proteins and inflammatory pathways:

Homozygous loss of function mutations in DSG1 caused severe skin dermatitis, multiple allergies, and metabolic wasting syndrome, referred to as SAM-syndrome. These patients suffer from severe food allergies, markedly elevated IgE levels and recurrent infections. Although the defective skin barrier certainly contributes to an inflammatory phenotype, cultured keratinocytes derived from these patients revealed increased mRNA levels of the cytokines thymic stromal lymphopoietin (TSLP), IL-5, and TNF-α in the absence of any inflammatory trigger suggesting a direct role of DSG1 in limiting inflammatory responses (Samuelov et al., 2013). Notably, DSG1 is also present in the esophageal epithelium. Its expression is decreased in esophageal biopsies from patients with eosinophilic esophagitis, an allergic disorder characterized by chronic inflammation of the esophageal mucosa. The gene expression profile from DSG1 knockdown esophageal epithelial cells substantially overlapped with the transcriptome of the inflamed esophageal mucosa from patients with eosinophilic esophagitis (Sherrill et al., 2014). Since DSG1 can suppress the MAPK pathway through EGFR signaling, and EGFR signaling has been shown to regulate key factors involved in skin inflammation, DSG1 might block inflammatory processes through suppression of EGFR/MAPK signaling (Lichtenberger et al., 2013). DSG4 is highly expressed in hair follicles and at a lower level in the granular layer of the human epidermis. Loss of function mutations in DSG4 led to hypotrichosis but some patients also developed erythema, scaling and skin erosions (Ullah et al., 2015). Treatment of DSG4-deficient rats with the TLR7 ligand imiquimod induced a skin inflammation with increased expression of the pro-inflammatory cytokines IL-1β and IL-8. Although the molecular mechanisms are unclear and experiments using isolated keratinocytes are missing, these data suggest a role for DSG4 in suppressing TLR-mediated inflammatory processes (Moreno-Sosa et al., 2021). In contrast to the suprabasal DSG1 and DSG4 isoforms, basally expressed DSG3 appeared to promote inflammation. This correlates with its role in promoting EGFR activation and proliferation (Ri et al., 2019). In an anaphylactic rhinitis model, silencing of DSG3 mediated inhibition of EGFR signaling and decreased TNF-α, IL-4, and IL-6 levels (Ri et al., 2019). Similarly, the knockdown of DSG3 decreased TNF-α, IL-6, and IL-8 levels in a mouse model for chronic rhinosinusitis, although in this case inhibition of Wnt signaling was considered as responsible for alleviating inflammation (Cheng et al., 2019).

DSG2 and DSC2, the primary isoforms in simple epithelia, are also expressed in the heart and at low amounts in the basal layer of stratified epithelia. Loss of function mutations affecting DSG2 and DSC2 result in heart defects and in the case of DSC2 in mild palmoplantar keratoderma, and wooly hair (Lee and McGrath, 2021). DSG2 appears to be involved in the pathogenesis of Crohn’s disease (CD), a type of inflammatory bowel disease, as it is strongly reduced in the mucosa of patients suffering from CD (Spindler et al., 2015). Intestine-specific DSG2 knockout mice developed a more-pronounced colitis after dextran sodium sulfate or *Citrobacter rodentium* exposure accompanied by the activation of epithelial pSTAT3 signaling and increased mRNA amounts of the pro-inflammatory cytokines IL-1β and TNF-α (Gross et al., 2018). The observation that DSG2 regulates p38MAPK activity in cultured enterocytes, as shown by RNAi and treatment with DSG2-inhibiting antibodies (Ungewiss et al., 2017), raises the possibility that DSG2 controls inflammatory processes through p38MAPK signaling.

Transgenic mice overexpressing DSC2 in cardiac myocytes developed severe cardiac dysfunction. Remarkably, gene expression analyses revealed an upregulation of several chemokines and chemokine receptors as well as interleukins and interleukin receptors, suggesting that DSC2 overexpression provoked an acute sterile cardiac inflammation (Brodehl et al., 2017). So far, no human disorder has been linked to DSC1 mutations. However, mice lacking DSC1 showed epidermal fragility, skin barrier defects and defective skin differentiation as well as chronic dermatitis. If disturbed signaling pathways in DSC1 knockout keratinocytes contributed to this inflammation remains to be determined (Chidgey et al., 2001). Mutation in the human DSC3 gene caused hypotrichosis, sometimes accompanied by skin fragility (Onoufriadis et al., 2020; Lee and McGrath, 2021). DSC3-deficient mice showed a pre-implantation lethal phenotype. However, severe skin fragility, telogen hair loss and massive inflammation was observed in mice lacking epidermal DSC3 (Chen et al., 2008) and knockout of DSC3 in IECs exacerbates azoxymethane and dextrane sodium sulfate induced ulcerative colitis (Ostermann et al., 2019). Thus, DSC3 may play a role in limiting inflammatory responses.

Mutations in the desmosomal plaque proteins PKP1, PKP2, PG and DSP cause severe diseases of the skin and/or the heart (Lee and McGrath, 2021). Again, disorders of the skin often go along with sustained inflammation. Known disorders caused by *PG* mutations affect the heart and the skin. However, the severity of skin disorders can vary from diffuse palmoplantar keratodermas and congenital wooly hair to fatal skin fragility resulting in lethal congenital epidermolysis bullosa (Lee and McGrath, 2021). The tissue specific knockout of PG in keratinocytes resulted in increased cornification, epidermal thickening, ulceration and inflammation (Li et al., 2012). Loss of function in murine cardiomyocytes recapitulated the symptoms of human ACM, including inflammation (Li D. et al., 2011; Li J. et al., 2011). However, these studies did not address if PG intrinsically regulates inflammatory responses. Although, the precise role of PG in Wnt signaling is still not fully understood, PG appears to be able to regulate context dependent transcriptional activity of TCF/LEF directly and indirectly (reviewed in Huber and Petersen, 2015; Aktary et al., 2017; Piven and Winata, 2017). Since Wnt signaling participates in the modulation of inflammatory cytokine production e.g., through NF-κB signaling and other innate defense mechanisms (Jridi et al., 2020), PG could regulate inflammatory processes through modulating Wnt signaling. Moreover, Spindler et al. (2014) demonstrated that depletion of PG in keratinocytes induced the activation of p38MAPK, which can be rescued by extranuclear PG expression, thus providing another putative link to inflammatory pathways.

PKP1, which is almost exclusively expressed in stratified epithelia, is indispensable for desmosomal cohesion *in vitro* and *in vivo* (McGrath et al., 1997; Tucker et al., 2014; Keil et al., 2016; Rietscher et al., 2016). Loss of function mutations of PKP1 cause the epidermal dysplasia skin fragility syndrome (EDSFS) characterized by severe skin erosions, dystrophic nails, sparse hair, and a painful debilitating thickening and cracking of the palms and soles. Moreover, generalized neonatal erythema, chronic perioral inflammation (cheilitis), recurrent skin infections and mild to severe pruritus were observed in the majority of cases (McGrath and Mellerio, 2010). An upregulation of PKP1 transcripts has been reported in prevalent skin diseases associated with inflammation and hyperproliferation, such as psoriasis (Kulski et al., 2005; Hatzfeld et al., 2014). So far, it is unclear if PKP1 intrinsically affects inflammatory responses.

Heterozygous loss of function mutations in PKP2 are the most common genetic cause of ACM (Gerull et al., 2004). Recent data suggest that inflammatory processes essentially participate in the progression of ACM. Intriguingly, in PKP2-deficient myocytes a large number of transcripts associated with inflammatory responses were upregulated (Perez-Hernandez et al., 2020). Consistently, PKP2 has been shown to regulate EGFR/p38MAPK and PKCα signaling pathways (Bass-Zubek et al., 2008; Arimoto et al., 2014; Dubash et al., 2016; Hao et al., 2019), which can affect the transcription of genes that are altered in PKP2 knockout cardiomyocytes.

For PKP3, no human disorder has been described so far. However, data from knockout animals suggest again a role for PKP3 in the regulation of inflammatory processes. PKP3 knockout mice suffered from defective local and systemic immune responses, at least partially mediated through a function of PKP3 in the hematopoietic system (Sklyarova et al., 2008, 2015). Like PKP2, PKP3 had an impact on ERK/p38MAPK signaling (Lim et al., 2019) and inflammation associated genes like IL-6, chemokine (C-C Motif) ligand 2 (CCL2), S100A8 and S100A9, were upregulated upon PKP3 knockdown in HaCaT and fetal buccal mucosal cell lines (Basu et al., 2015).

DSP is present in all desmosome bearing tissues. Loss of function mutations cause a variety of diseases affecting the heart and/or the skin. Several of these disorders are accompanied by dysregulated inflammation and/or immune response (Najor, 2018; Lee and McGrath, 2021). In analogy to DSG1, DSP loss of function mutation can cause the SAM-syndrome (McAleer et al., 2015). Moreover, recent reports indicate that myocardial inflammation is an important factor in the development and progression of DSP-associated cardiomyopathy (Reichl et al., 2018; Protonotarios et al., 2019; Smith et al., 2020). Mechanistically, DSP has been shown to regulate ERK/p38MAPK and Wnt signaling in several cell lines and animal models (Yang et al., 2012; Martherus et al., 2016; Kam et al., 2018; Bendrick et al., 2019), suggesting a role of DSP-dependent signaling in inflammation and immune responses.

**FIGURE 5:**
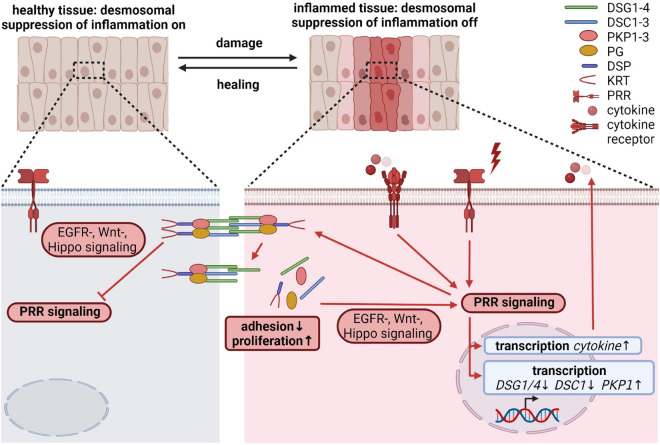
Desmosomal proteins regulate inflammatory processes during wound healing *(created with biorender.com)*. Activated PRR signaling through intrinsic or extrinsic insults as well as upon tissue wounding induce signaling cascades affecting the amount and/or the localization of desmosomal proteins and thus cellular cohesion and proliferation. Moreover, through interfering with various signaling pathways including p38MAPK, Wnt, and Hippo signaling, desmosomal proteins are able to modulate inflammatory responses. Since most desmosomal proteins have been described to dampen inflammation, these proteins could be required to locally restrict inflammatory responses and/or ensure the resolution of inflammatory responses required for tissue regeneration.

Taken together, several lines of evidence suggest a role of desmosomal proteins in regulating inflammatory processes in wounded tissues or upon barrier disturbance. The same processes that shift desmosomal adhesion from the hyperadhesive to the dynamic state might induce PTMs in desmosomal proteins enabling them to monitor inflammatory processes. With the exception of DSG3 and DSC2 all desmosomal proteins have been described to repress inflammatory responses. The resolution of inflammation is an active process responsible for switching inflammation off. This process is essential to fully restore tissue function but is so far only incompletely understood (Feehan and Gilroy, 2019). Current knowledge supports the hypothesis that the resolution phase might critically depend on desmosomal proteins (Figure 5). Elucidating the underlying molecular mechanisms might facilitate the development of therapies for chronic wounds as well as inflammatory skin diseases.

## Future Perspectives

Several recurring trends arise throughout the studies on desmosomal proteins in cell signaling: The desmosomal cadherins affect mitogenic signaling primarily by controlling EGFR activity. While suprabasally expressed protein isotypes typically dampen the activation of EGFR induced kinase cascades, those desmosomal cadherins that are expressed in proliferating basal cells rather promote EGFR signaling. The function and regulation of the plaque proteins is more complex and only partially understood. These proteins are targets of various chemical and mechanical stimuli and are strongly modified by posttranslational modifications, particularly phosphorylation. They are essential for intercellular cohesion but have a number of extradesmosomal functions in Wnt, Hippo, EGF and IGF1/insulin signaling. Downstream of these signals, the PKPs control RNA metabolism including protein translation. However, the role of extradesmosomal DSP is largely unknown despite a considerable cytoplasmic pool. Future studies need to characterize those functions to fully understand the role of desmosomal proteins in coordinating proliferation, differentiation and CIP as well as in inflammation. This is a prerequisite to understand their context-dependent role in carcinoma development and progression and in wound healing. So far, most studies focus on a single desmosomal protein to elucidate its function in cell adhesion and in signaling. However, activation of signaling pathways leads to modifications not only of a single protein but has far-reaching effects. Thus, a future challenge is to analyze and manipulate native desmosomal protein complexes and look at these proteins at once to define their role within the junctional network and understand how desmosomal and extradesmosomal functions are coordinated.

## Author Contributions

All authors conceived and wrote the manuscript and designed the figures.

## Conflict of Interest

The authors declare that the research was conducted in the absence of any commercial or financial relationships that could be construed as a potential conflict of interest.

## Publisher’s Note

All claims expressed in this article are solely those of the authors and do not necessarily represent those of their affiliated organizations, or those of the publisher, the editors and the reviewers. Any product that may be evaluated in this article, or claim that may be made by its manufacturer, is not guaranteed or endorsed by the publisher.

## Footnotes

1
http://clipdb.ncrnalab.org
