# Supplementary material for: Desmosomes as Signaling Hubs in the Regulation of Cell Behavior
Source: Front Cell Dev Biol. 2021 Sep 23;9:745670. doi: 10.3389/fcell.2021.745670 (PMC8495202; doi:10.3389/fcell.2021.745670)
Supplement: Supplementary file 1 [file Table_1.DOCX]

**Table S1: Overview of mouse models for desmosomal proteins.**

Listed are general (g) and tissue specific (c = cardiac, iec = intestinal epithelial cells, epi = epidermal, hem = hematopoetic, corn = cornea, ind = inducible) knockout (KO) approaches as well as transgenic mouse models with tissue specific transgene expression (Krt1, Inv 🡪 suprabasal keratinocytes; Krt5, Krt14 🡪 basal keratinocytes; Myh6 🡪 cardiac myocytes). Transgenic mouse models concerning disease causing point mutations (Mohammed and Chidgey, 2021) have not been considered and are reviewed elsewhere (Gerull and Brodehl, 2020). Phenotype terms dealing with the focus of our review are highlighted in bold.

ACM, arrhythmogenic cardiomyopathy; AOM, azoxymethane; DSS, dextrane sodium sulfate; n.d., not determined; LPS, lipopolysaccharide; KD, knockdown PMA, Phorbol-12-myristat-13-acetat; PV, Pemphigus vulgaris

| **Gene** | **Mouse model** | **Phenotype** | **Signaling pathway** | **References** |
| --- | --- | --- | --- | --- |
| DSG1 | *Dsg1^g_KO^* | perinatal lethal, epidermal water loss, severe blister formation | n.d. | (Kugelmann et al., 2019) |
|  | Krt5:mDsg1α-Etag | no apparent effect on skin, mucous membranes, hair follicles, and other tissues | n.d. | (Hata et al., 2011) |
| DSG2 | *Dsg2^g_KO^* | embryonic lethality, **decreased stem cell proliferation** | n.d. | (Eshkind et al., 2002) |
|  | *Dsg2^c_KO^* | development of ACM, **increased cardiac inflammation** | n.d. | (Kant et al., 2015) |
|  | *Dsg2^ΔE4-6^* | development of ACM, **increased cardiac inflammation** | n.d. | (Krusche et al., 2011; Lubos et al., 2020) |
|  | *Dsg2^iec_KO^* | disturbed intestinal barrier, **more-pronounced DSS- and Citrobacter rodentium-induced colitis** | increased STAT3 signaling upon challenging | (Gross et al., 2018) |
|  | Myh6:mDsg2-Flag | no phenotype | n.d. | (Pilichou et al., 2006) |
|  | Inv:mDsg2-Flag | **extensive epidermal hyperplasia and increased keratinocyte proliferation *in vivo* and *in vitro*** | increased PI3K/AKT, MEK-MAPK, STAT3 and NFκB signaling | (Brennan et al., 2007) |
|  | Krt14:mDsg2-Flag | **accelerated cutaneous wound healing** and **increased** **wound-adjacent keratinocyte proliferation** | increased EGFR/MAPK, PI3K/AKT, c‑Src and STAT3 signaling | (Cooper et al., 2018) |

| **Gene** | **Mouse model** | **Phenotype** | **Signaling pathway** | **References** |
| --- | --- | --- | --- | --- |
| DSG3 | *Dsg3^g_KO^* | suprabasilar acantholysis as seen in PV, hair loss, **inflammatory oral erosion** | n.d. | (Koch et al., 1997) |
|  | Krt14:Flag-hDsg3Δaa72-569 | **hyperproliferation of keratinocytes**, abnormal differentiation, **inflammation** | n.d. | (Allen et al., 1996) |
|  | Inv:mDSG3-Flag | postnatal lethal due to severe dehydration, structural alteration of the stratum corneum | n.d. | (Elias et al., 2001) |
|  | Krt1:hDsg3 | **epidermal hyperproliferation**, abnormal differentiation, features of **chronic dermatitis** and ichthyosis | n.d. | (Merritt et al., 2002) |
|  | siRNA mediated KD of DSG3 in nasal mucosa tissue | **inhibition of inflammation** in a chronic rhinosinusitis mouse model | decreased Wnt signaling | (Cheng et al., 2019) |
|  | siRNA mediated KD of DSG3 in nasal mucosa tissue | **decreased inflammation** in a anaphylactic rhinitis mouse model | decreased EGFR activation | (Ri et al., 2019) |
| DSC1 | *Dsc1^g_KO^* | epidermal fragility, barrier defects, **epidermal hyperproliferation**, abnormal differentiation, **chronic dermatitis** | n.d. | (Chidgey et al., 2001) |
|  | *Dsc1^ΔE17^* | no phenotype | n.d. | (Cheng et al., 2004) |
|  | Krt14:hDsc1a | no phenotype | n.d. | (Henkler et al., 2001) |
| DSC2 | *Dsc2^g_KO^* | decreased cardiac stress resistance | PI3K/AKT and MAPK signaling unaffected | (Rimpler, 2014) |
|  | *Dsc2^iec_KO^* | no phenotype | diminished EGFR and p38MAPK activation upon DSS treatment | (Gross et al., 2018) |
|  | *Dsc2^ind_iec_KO^* | impaired mucosal repair after biopsy-induced wounding and **impaired** **recovery from DSS-induced colitis**, increased intestinal permeability | n.d. | (Flemming et al., 2020) (Raya-Sandino et al., 2021) |
|  | Myh6:hDsc2-HA | severe cardiac dysfunction, **upregulation of inflammation, and fibrosis related genes** | n.d. | (Brodehl et al., 2017) |
| **Gene** | **Mouse model** | **Phenotype** | **Signaling pathway** | **References** |
| DSC3 | *Dsc3^g_KO^* | pre-implantation lethality | n.d. | (Den et al., 2006) |
|  | *Dsc3^epi_KO^* | intraepidermal skin blistering and telogen hair loss, severe epidermal hyperplasia, **increased basal cell proliferation**, **lesions with massive inflammation** and scar formation | n.d. | (Chen et al., 2008) |
|  | *Dsc3^iec_KO^* | **increased ulcerative colitis** and elevated tumour burden upon AOM and DSS application | n.d. | (Ostermann et al., 2019) |
|  | Krt1:mDsc3a + Krt1:mDsc3b | variable ventral alopecia, acanthosis, hypergranulosis and hyperkeratosis, **increased basal and suprabasal keratinocyte proliferation** | increased Wnt signaling in primary keratinocytes | (Hardman et al., 2005) |
| JUP | *JUP^g_KO^* | embryonic lethality due to cardiac defects | n.d. | (Ruiz et al., 1996) |
|  | *JUP^g_KO^*C57Bl/6 background | perinatal lethality, cardiac dysfunction, skin blistering, subcorneal acantholysi | n.d. | (Bierkamp et al., 1996) |
|  | *JUP^c_KO^* | development of ACM, **increased inflammation** and fibrosis | Wnt, p38MAPK and JNK signaling unaffected, increased TGFβ signaling | (Li et al., 2011a) |
|  | *JUP^ind_c_KO^* | progressive development of ACM, **increased inflammation** and fibrosis | increased Wnt and Akt signaling | (Li et al., 2011b) |
|  | *JUP^epi_KO^* | overcornification and thickening of the epidermis, skin ulceration and **inflammation**, increased apoptosis and **proliferation of keratinocytes** | Wnt signaling unaffected | (Li et al., 2012) |
|  | *JUP^corn_KO^* | impaired tissue integrity, **wound healing** and differentiation | n.d. | (Kokado et al., 2018) |
| PKP1 | *PKP1^g_KO^* | neonatal lethality, skin fragility, disturbed barrier, growth retardation | n.d. | (Rietscher et al., 2018) |
| PKP2 | *PKP2^g_KO^* | mid-gestational embryonic lethality due to cardiac defects | n.d. | (Grossmann et al., 2004) |
|  | *PKP2^ind_c_KO^* | development of ACM with extensive fibrosis and heart failure, **upregulation of inflammation, immune response and fibrosis related genes** | n.d. | (Cerrone et al., 2017; Perez-Hernandez et al., 2020) |
| **Gene** | **Mouse model** | **Phenotype** | **Signaling pathway** | **References** |
| PKP3 | *PKP3^g_KO^* | defective hair follicle morphogenesis, **increased keratinocyte proliferation**, **increased susceptibility to dermatitis** | n.d. | (Sklyarova et al., 2008) |
|  | *PKP3^hem_KO^* | **increased susceptibility to PMA-induced dermatitis, DSS-induced colitis and LPS-induced systemic inflammation** | n.d. | (Sklyarova et al., 2015) |
| DSP | *DSP^g_KO^* | embryonic lethality | n.d. | (Gallicano et al., 1998) |
|  | *DSP^c_KO^* | high embryonic lethality and postnatal lethality of surviving embryos due to cardiac defects | suppressed Wnt signaling | (Garcia-Gras et al., 2006) |
|  | *DSP^epi_KO^* | mechanical stress induced intercellular separations, defective cell-cell adhesion and cytoskeletal organization | n.d. | (Vasioukhin et al., 2001) |
|  | *DSP^iec_KO^* | microvilli defects, intercellular adhesion was not affected | n.d. | (Sumigray and Lechler, 2012) |

**References**

Allen, E., Yu, Q.C., and Fuchs, E. (1996). Mice expressing a mutant desmosomal cadherin exhibit abnormalities in desmosomes, proliferation, and epidermal differentiation. *J Cell Biol* 133(6)**,** 1367-1382. doi: 10.1083/jcb.133.6.1367.

Bierkamp, C., McLaughlin, K.J., Schwarz, H., Huber, O., and Kemler, R. (1996). Embryonic heart and skin defects in mice lacking plakoglobin. *Dev Biol* 180(2)**,** 780-785. doi: 10.1006/dbio.1996.0346.

Brennan, D., Hu, Y., Joubeh, S., Choi, Y.W., Whitaker-Menezes, D., O'Brien, T., et al. (2007). Suprabasal Dsg2 expression in transgenic mouse skin confers a hyperproliferative and apoptosis-resistant phenotype to keratinocytes. *J Cell Sci* 120(Pt 5)**,** 758-771. doi: 10.1242/jcs.03392.

Brodehl, A., Belke, D.D., Garnett, L., Martens, K., Abdelfatah, N., Rodriguez, M., et al. (2017). Transgenic mice overexpressing desmocollin-2 (DSC2) develop cardiomyopathy associated with myocardial inflammation and fibrotic remodeling. *PLoS One* 12(3)**,** e0174019. doi: 10.1371/journal.pone.0174019.

Cerrone, M., Montnach, J., Lin, X., Zhao, Y.T., Zhang, M., Agullo-Pascual, E., et al. (2017). Plakophilin-2 is required for transcription of genes that control calcium cycling and cardiac rhythm. *Nat Commun* 8(1)**,** 106. doi: 10.1038/s41467-017-00127-0.

Chen, J., Den, Z., and Koch, P.J. (2008). Loss of desmocollin 3 in mice leads to epidermal blistering. *J Cell Sci* 121(Pt 17)**,** 2844-2849. doi: 10.1242/jcs.031518.

Cheng, J., Yang, J., Xue, K., Zhao, Y., Zhao, C., Li, S., et al. (2019). Desmoglein 3 Silencing Inhibits Inflammation and Goblet Cell Mucin Secretion in a Mouse Model of Chronic Rhinosinusitis via Disruption of the Wnt/beta-Catenin Signaling Pathway. *Inflammation* 42(4)**,** 1370-1382. doi: 10.1007/s10753-019-00998-z.

Cheng, X., Mihindukulasuriya, K., Den, Z., Kowalczyk, A.P., Calkins, C.C., Ishiko, A., et al. (2004). Assessment of splice variant-specific functions of desmocollin 1 in the skin. *Mol Cell Biol* 24(1)**,** 154-163. doi: 10.1128/MCB.24.1.154-163.2004.

Chidgey, M., Brakebusch, C., Gustafsson, E., Cruchley, A., Hail, C., Kirk, S., et al. (2001). Mice lacking desmocollin 1 show epidermal fragility accompanied by barrier defects and abnormal differentiation. *J Cell Biol* 155(5)**,** 821-832. doi: 10.1083/jcb.200105009.

Cooper, F., Overmiller, A.M., Loder, A., Brennan-Crispi, D.M., McGuinn, K.P., Marous, M.R., et al. (2018). Enhancement of Cutaneous Wound Healing by Dsg2 Augmentation of uPAR Secretion. *J Invest Dermatol* 138(11)**,** 2470-2479. doi: 10.1016/j.jid.2018.04.024.

Den, Z., Cheng, X., Merched-Sauvage, M., and Koch, P.J. (2006). Desmocollin 3 is required for pre-implantation development of the mouse embryo. *J Cell Sci* 119(Pt 3)**,** 482-489. doi: 10.1242/jcs.02769.

Elias, P.M., Matsuyoshi, N., Wu, H., Lin, C., Wang, Z.H., Brown, B.E., et al. (2001). Desmoglein isoform distribution affects stratum corneum structure and function. *J Cell Biol* 153(2)**,** 243-249. doi: 10.1083/jcb.153.2.243.

Eshkind, L., Tian, Q., Schmidt, A., Franke, W.W., Windoffer, R., and Leube, R.E. (2002). Loss of desmoglein 2 suggests essential functions for early embryonic development and proliferation of embryonal stem cells. *Eur J Cell Biol* 81(11)**,** 592-598. doi: 10.1078/0171-9335-00278.

Flemming, S., Luissint, A.C., Kusters, D.H.M., Raya-Sandino, A., Fan, S., Zhou, D.W., et al. (2020). Desmocollin-2 promotes intestinal mucosal repair by controlling integrin-dependent cell adhesion and migration. *Mol Biol Cell* 31(6)**,** 407-418. doi: 10.1091/mbc.E19-12-0692.

Gallicano, G.I., Kouklis, P., Bauer, C., Yin, M., Vasioukhin, V., Degenstein, L., et al. (1998). Desmoplakin is required early in development for assembly of desmosomes and cytoskeletal linkage. *J Cell Biol* 143(7)**,** 2009-2022. doi: 10.1083/jcb.143.7.2009.

Garcia-Gras, E., Lombardi, R., Giocondo, M.J., Willerson, J.T., Schneider, M.D., Khoury, D.S., et al. (2006). Suppression of canonical Wnt/beta-catenin signaling by nuclear plakoglobin recapitulates phenotype of arrhythmogenic right ventricular cardiomyopathy. *J Clin Invest* 116(7)**,** 2012-2021. doi: 10.1172/JCI27751.

Gerull, B., and Brodehl, A. (2020). Genetic Animal Models for Arrhythmogenic Cardiomyopathy. *Front Physiol* 11**,** 624. doi: 10.3389/fphys.2020.00624.

Gross, A., Pack, L.A.P., Schacht, G.M., Kant, S., Ungewiss, H., Meir, M., et al. (2018). Desmoglein 2, but not desmocollin 2, protects intestinal epithelia from injury. *Mucosal Immunol* 11(6)**,** 1630-1639. doi: 10.1038/s41385-018-0062-z.

Grossmann, K.S., Grund, C., Huelsken, J., Behrend, M., Erdmann, B., Franke, W.W., et al. (2004). Requirement of plakophilin 2 for heart morphogenesis and cardiac junction formation. *J Cell Biol* 167(1)**,** 149-160. doi: 10.1083/jcb.200402096.

Hardman, M.J., Liu, K., Avilion, A.A., Merritt, A., Brennan, K., Garrod, D.R., et al. (2005). Desmosomal cadherin misexpression alters beta-catenin stability and epidermal differentiation. *Mol Cell Biol* 25(3)**,** 969-978. doi: 10.1128/MCB.25.3.969-978.2005.

Hata, T., Nishifuji, K., Shimoda, K., Sasaki, T., Yamada, T., Nishikawa, T., et al. (2011). Transgenic rescue of desmoglein 3 null mice with desmoglein 1 to develop a syngeneic mouse model for pemphigus vulgaris. *J Dermatol Sci* 63(1)**,** 33-39. doi: 10.1016/j.jdermsci.2011.04.010.

Henkler, F., Strom, M., Mathers, K., Cordingley, H., Sullivan, K., and King, I. (2001). Trangenic misexpression of the differentiation-specific desmocollin isoform 1 in basal keratinocytes. *J Invest Dermatol* 116(1)**,** 144-149. doi: 10.1046/j.1523-1747.2001.00234.x.

Kant, S., Holthofer, B., Magin, T.M., Krusche, C.A., and Leube, R.E. (2015). Desmoglein 2-Dependent Arrhythmogenic Cardiomyopathy Is Caused by a Loss of Adhesive Function. *Circ Cardiovasc Genet* 8(4)**,** 553-563. doi: 10.1161/CIRCGENETICS.114.000974.

Koch, P.J., Mahoney, M.G., Ishikawa, H., Pulkkinen, L., Uitto, J., Shultz, L., et al. (1997). Targeted disruption of the pemphigus vulgaris antigen (desmoglein 3) gene in mice causes loss of keratinocyte cell adhesion with a phenotype similar to pemphigus vulgaris. *J Cell Biol* 137(5)**,** 1091-1102. doi: 10.1083/jcb.137.5.1091.

Kokado, M., Miyajima, M., Okada, Y., Ichikawa, K., Yamanaka, O., Liu, C.Y., et al. (2018). Lack of plakoglobin impairs integrity and wound healing in corneal epithelium in mice. *Lab Invest* 98(11)**,** 1375-1383. doi: 10.1038/s41374-018-0082-z.

Krusche, C.A., Holthofer, B., Hofe, V., van de Sandt, A.M., Eshkind, L., Bockamp, E., et al. (2011). Desmoglein 2 mutant mice develop cardiac fibrosis and dilation. *Basic Res Cardiol* 106(4)**,** 617-633. doi: 10.1007/s00395-011-0175-y.

Kugelmann, D., Radeva, M.Y., Spindler, V., and Waschke, J. (2019). Desmoglein 1 Deficiency Causes Lethal Skin Blistering. *J Invest Dermatol* 139(7)**,** 1596-1599 e1592. doi: 10.1016/j.jid.2019.01.002.

Li, D., Liu, Y., Maruyama, M., Zhu, W., Chen, H., Zhang, W., et al. (2011a). Restrictive loss of plakoglobin in cardiomyocytes leads to arrhythmogenic cardiomyopathy. *Hum Mol Genet* 20(23)**,** 4582-4596. doi: 10.1093/hmg/ddr392.

Li, D., Zhang, W., Liu, Y., Haneline, L.S., and Shou, W. (2012). Lack of plakoglobin in epidermis leads to keratoderma. *J Biol Chem* 287(13)**,** 10435-10443. doi: 10.1074/jbc.M111.299669.

Li, J., Swope, D., Raess, N., Cheng, L., Muller, E.J., and Radice, G.L. (2011b). Cardiac tissue-restricted deletion of plakoglobin results in progressive cardiomyopathy and activation of {beta}-catenin signaling. *Mol Cell Biol* 31(6)**,** 1134-1144. doi: 10.1128/MCB.01025-10.

Lubos, N., van der Gaag, S., Gercek, M., Kant, S., Leube, R.E., and Krusche, C.A. (2020). Inflammation shapes pathogenesis of murine arrhythmogenic cardiomyopathy. *Basic Res Cardiol* 115(4)**,** 42. doi: 10.1007/s00395-020-0803-5.

Merritt, A.J., Berika, M.Y., Zhai, W., Kirk, S.E., Ji, B., Hardman, M.J., et al. (2002). Suprabasal desmoglein 3 expression in the epidermis of transgenic mice results in hyperproliferation and abnormal differentiation. *Mol Cell Biol* 22(16)**,** 5846-5858. doi: 10.1128/MCB.22.16.5846-5858.2002.

Mohammed, F., and Chidgey, M. (2021). Desmosomal protein structure and function and the impact of disease-causing mutations. *J Struct Biol* 213(3)**,** 107749. doi: 10.1016/j.jsb.2021.107749.

Ostermann, A.L., Wunderlich, C.M., Schneiders, L., Vogt, M.C., Woeste, M.A., Belgardt, B.F., et al. (2019). Intestinal insulin/IGF1 signalling through FoxO1 regulates epithelial integrity and susceptibility to colon cancer. *Nat Metab* 1(3)**,** 371-389. doi: 10.1038/s42255-019-0037-8.

Perez-Hernandez, M., Marron-Linares, G.M., Schlamp, F., Heguy, A., van Opbergen, C.J.M., Mezzano, V., et al. (2020). Transcriptomic Coupling of PKP2 With Inflammatory and Immune Pathways Endogenous to Adult Cardiac Myocytes. *Front Physiol* 11**,** 623190. doi: 10.3389/fphys.2020.623190.

Pilichou, K., Nava, A., Basso, C., Beffagna, G., Bauce, B., Lorenzon, A., et al. (2006). Mutations in desmoglein-2 gene are associated with arrhythmogenic right ventricular cardiomyopathy. *Circulation* 113(9)**,** 1171-1179. doi: 10.1161/CIRCULATIONAHA.105.583674.

Raya-Sandino, A., Luissint, A.C., Kusters, D.H.M., Narayanan, V., Flemming, S., Garcia-Hernandez, V., et al. (2021). Regulation of intestinal epithelial intercellular adhesion and barrier function by desmosomal cadherin desmocollin-2. *Mol Biol Cell* 32(8)**,** 753-768. doi: 10.1091/mbc.E20-12-0775.

Ri, H., Peiyan, Z., Jianqi, W., Yunteng, Z., Gang, L., and Baoqing, S. (2019). Desmoglein 3 gene mediates epidermal growth factor/epidermal growth factor receptor signaling pathway involved in inflammatory response and immune function of anaphylactic rhinitis. *Biomed Pharmacother* 118**,** 109214. doi: 10.1016/j.biopha.2019.109214.

Rietscher, K., Keil, R., Jordan, A., and Hatzfeld, M. (2018). 14-3-3 proteins regulate desmosomal adhesion via plakophilins. *J Cell Sci* 131(10). doi: 10.1242/jcs.212191.

Rimpler, U. (2014). *Funktionelle Charakterisierung von Desmocollin 2 während der Embryonalentwicklung und im adulten Herzen in der Maus.* [dissertation]. Humboldt-Universität zu Berlin, Mathematisch-Naturwissenschaftliche Fakultät I.

Ruiz, P., Brinkmann, V., Ledermann, B., Behrend, M., Grund, C., Thalhammer, C., et al. (1996). Targeted mutation of plakoglobin in mice reveals essential functions of desmosomes in the embryonic heart. *J Cell Biol* 135(1)**,** 215-225. doi: 10.1083/jcb.135.1.215.

Sklyarova, T., Bonne, S., D'Hooge, P., Denecker, G., Goossens, S., De Rycke, R., et al. (2008). Plakophilin-3-deficient mice develop hair coat abnormalities and are prone to cutaneous inflammation. *J Invest Dermatol* 128(6)**,** 1375-1385. doi: 10.1038/sj.jid.5701189.

Sklyarova, T., van Hengel, J., Van Wonterghem, E., Libert, C., van Roy, F., and Vandenbroucke, R.E. (2015). Hematopoietic plakophilin-3 regulates acute tissue-specific and systemic inflammation in mice. *Eur J Immunol* 45(10)**,** 2898-2910. doi: 10.1002/eji.201445440.

Sumigray, K.D., and Lechler, T. (2012). Desmoplakin controls microvilli length but not cell adhesion or keratin organization in the intestinal epithelium. *Mol Biol Cell* 23(5)**,** 792-799. doi: 10.1091/mbc.E11-11-0923.

Vasioukhin, V., Bowers, E., Bauer, C., Degenstein, L., and Fuchs, E. (2001). Desmoplakin is essential in epidermal sheet formation. *Nat Cell Biol* 3(12)**,** 1076-1085. doi: 10.1038/ncb1201-1076.
